# Quantifying the Multidimensional Impact of Cyber Attacks in Digital Financial Services: A Systematic Literature Review

**DOI:** 10.3390/s25144345

**Published:** 2025-07-11

**Authors:** Olumayowa Adefowope Adekoya, Hany F. Atlam, Harjinder Singh Lallie

**Affiliations:** Cyber Security Centre, Warwick Manufacturing Group, University of Warwick, Coventry CV4 7AL, UK; olumayowa.adekoya@warwick.ac.uk (O.A.A.);

**Keywords:** risk quantification, impact quantification, taxonomy, cyber security impact, multidimensional impact, digital financial service, machine learning, deep learning

## Abstract

The increasing frequency and sophistication of cyber attacks have posed significant challenges for digital financial organisations, particularly in quantifying their multidimensional impacts. These challenges are largely attributed to the lack of a standardised cyber impact taxonomy, limited data availability, and the evolving nature of technological threats. As a result, organisations often struggle with ineffective security investment prioritisation, reactive incident response planning, and the inability to implement robust, risk-based controls. Hence, an efficient and comprehensive approach is needed to quantify the diverse impacts of cyber attacks in digital financial services. This paper presents a systematic review and examination of the state of the art in cyber impact quantification, with a particular focus on digital financial organisations. Based on a structured search strategy, 44 articles (out of 637) were selected for in-depth analysis. The review investigates the terminologies used to describe cyber impacts, categorises current quantification techniques (pre-attack and post-attack), and identifies the most commonly utilised internal and external data sources. Furthermore, it explores the application of Machine Learning (ML) and Deep Learning (DL) techniques in cyber security risk quantification. Our findings reveal a significant lack of standardised taxonomy for describing and quantifying the multidimensional impact of cyberattacks across physical, digital, economic, psychological, reputational, and societal dimensions. Lastly, open issues and future research directions are discussed. This work provides insights for researchers and professionals by consolidating and identifying quantification technique gaps in cyber security risk quantification.

## 1. Introduction

The digital financial services landscape is experiencing a significant revolution driven by increased customer adoption and technological innovation. In the United Kingdom, 86% of adults used online banking in 2024 [1]. Out of which 54% [1] used their mobile banking application as their primary channel to engage with their banks [2]. Global, digital payment transaction witnessing a 7% annual growth rate, with 3.4 trillion transactions processed in 2023 [3]. The financial technology (fintech) sector anticipates a potential global rebound in 2025, marked by a 15% growth in venture capital investment between the third and fourth quarters of 2024 [4]. There is a surge of new crypto currencies [5], and robo-advisors are experiencing rapid growth [4]. These figures underscore the unprecedented expansion and reliance on digital platforms within the financial sector. This digital transformation has undoubtedly ushered in human-like, connected, and empowering digital experiences [2] and efficiency in money management for individuals and businesses [6].

However, this convenience has increased reliance on digital services. The increased reliance on digital services comes with an expanded cyber security attack surface for malicious actors [7]. This expanded attack surface has led to a rise in cyber attacks. Consequently, the global average cost of a data breach increased by 10 % to 4.88 million dollars in 2024, according to IBM’s Data Breach Cost Report [8]. For the financial services industry, digital revolution has led to a serious surge in cyber attacks. Specifically, the World Economic Forum (WEF) reports that over 20,000 attacks resulted in 12 billion dollars in losses between 2004 and 2023 [9], highlighting the financial services’ disproportionate cyber attack and the significant risk to global financial stability.

Digital services and technology comprise various components and layers that work together to provide functionality, connectivity, and an excellent user experience, all of which we enjoy and provide us with a better quality of life. These digital services components and layers comprise numerous hardware devices, software, network devices, technology infrastructure, and databases. Oftentimes, the elements that make up these components have security vulnerabilities due to human errors, the complexity of software, the changing threat landscape, third-party components, and legacy code base, making them exploitable by cyber threat actors. For instance, the evolving landscape of Digital Twins in cyber–physical systems necessitates specialised risk auditing to address their inherent vulnerabilities [10]. These actors can exploit weaknesses in digital systems, targeting digital financial services providers in various ways to realise their malicious goals. According to the UK National Cyber Strategy [11], these malicious actors include nation-states, criminal groups, and hacktivists, and their most common cyber security attacks in 2024 were ransomware, phishing, and social engineering [12]. The detection of such evolving threats, including Android malware, increasingly relies on sophisticated Machine Learning approaches [13].

The cyber security impact of these attacks is not limited to financial losses alone as they also have a multidimensional impact, eroding trust, damaging reputations, and disrupting the smooth functioning of the entire financial ecosystem [14]. A comprehensive understanding of the multidimensional impact is not just crucial; it is imperative for digital financial services providers to develop effective strategies to manage their cyber security risk. It is critical to accurately quantify the potential impact of cyber security attacks before they occur so that organisations can prioritise security investments, develop effective incident response plans and build appropriate risk-balanced controls. This quantification, however, does not operate in a vacuum. It is integral to a holistic cyber security risk management process, encompassing identifying, assessing, treating, and monitoring cyber risks. A well-defined cyber risk management framework provides the context and strategic direction for meaningful risk quantification, ensuring that the results inform actionable mitigation and investment decisions. Effectively quantifying of these multidimensional cyber impacts requires a top-down approach, with active involvement and ownership from major organisational stakeholders. Senior leadership, including board members and C-suite executives, play a vital role as they are accountable and responsible for cyber resilience within the organisation. Their understanding and contextualisation of the potential financial, reputational, and operational consequences of cyberattacks which is informed by robust impact quantification is essential for making informed decisions on security investments, regulatory compliance, and incident response strategies. Furthermore, Artificial Intelligence (AI), specifically Machine Learning (ML) and Deep Learning (DL) offers a promising solution in this research area. ML and DL can analyse the complex, multidimensional financial data generated by cyber attacks, encompassing not only the direct costs but also the indirect impacts like reputational damage and operational disruption. ML and DL models have demonstrated exceptional capability in analysing vast amounts of data to identify patterns and predict future events [15].

This paper aims to provide a comprehensive investigation of the current landscape of quantifying the multidimensional impact of cyber attacks in digital financial services, including the implementation of ML and DL. By systematically reviewing state-of-the-art approaches, this paper identifies the most commonly used cyber attack impact taxonomy, the quantification techniques; datasets frequently used and applications of ML and DL in implementing cyber security risk quantification. This paper also explores the challenges of utilising ML and DL to quantify the multidimensional impact of cyber attacks and proposes potential solutions to overcome these barriers. Lastly, open issues and future research directions in quantifying the multidimensional impact of cyber attacks are presented. Through this systematic review, this paper contributes valuable guidance for future research and the development of more robust, efficient, and accurate techniques for quantifying the multidimensional impact of cyber attacks using ML and DL. Compared to similar reviews [16], this paper makes a distinct contribution by focusing specifically on the multidimensional impact of cyber attacks, their taxonomy, and the quantification of the different multidimensional impacts. While other reviews cover the broader quantification of cyber security risks, this paper narrows its scope to multidimensional cyber security impacts, their full spectrum, quantification techniques, and the application of ML and DL.

The contributions of this paper can be summarised as follows:

- Conducting a comprehensive investigation of the current landscape of the multidimensional cyber security impact taxonomies.
- Identifying the most common techniques used to quantify the multidimensional impact of cyber security attacks.
- Examining the most commonly used datasets in quantifying the impact of cyber security attacks.
- Identifying the most commonly used ML and DL techniques and their evaluation criteria for different implementations within the cyber security risk quantification research area.

The rest of this paper is organised as follows: Section 2 gives an overview of quantifying the multidimensional impact of cyber attacks; Section 3 introduces the research methodology that was adopted to conduct this review; Section 4 presents the analysis of the results; Section 5 provides the results and discussion, with the answers to research questions presented in detail; Section 6 presents open issues and future research directions regarding quantification of the multidimensional impact of cyber attacks; and Section 7 provides the conclusions.

## 2. An Overview of Quantifying the Multidimensional Impact of Cyber Attacks

This section provides an overview of quantifying the multidimensional impact of cyber attacks within digital financial services. It starts by exploring the need within digital financial services, assessing the established taxonomy, the techniques used for quantification, and the application of ML and DL in cyber security risk quantification. It also highlights how robust cyber risk management processes provide the foundational structure for effective quantification, ensuring that identified risks are measured and strategically addressed.

### 2.1. Digital Financial Services

The World Bank defines digital financial services as services that rely on digital technologies for consumer delivery and utilisation [17]. This definition’s digital technology delivery model aligns with the International Monetary Fund (IMF) [18], European Parliamentary Research Service [19] and Himanshu et al. [20] definition. These definitions highlights that digital financial services are a fundamental shift from traditional, physical, financial transactions to digitally mediated ones. This evidences that the digital transformation of finance services is directly linked to advancements in technologies like mobile devices, internet connectivity, and digital platforms [17]. These definitions also reveal two key goals of financial inclusion and economic development. Digital financial services can reach underserved populations, which is vital in developing economies. Likewise, where traditional banking is limited, digital financial services can drive economic growth, reduce transaction costs, and promote entrepreneurship. The most common examples of digital financial services activities and products include electronic money, digital wallets, and digital payment platforms [17], crypto-assets [19], remittances, and credit [18].

The delivery of digital financial services, including online banking, mobile banking, digital payment applications trading, and insurance platforms, relies on robust information technology infrastructure. Consequently, these information technology infrastructures’ design, development, and operational maintenance necessitate robustly managing evolving cyber security risks. This includes a detailed analysis of cybersecurity risks and threats inherent in such IT infrastructure, often guided by established frameworks like NIST [21]. This management is not merely reactive; it requires proactive identification, assessment, and treatment of potential threats. Cyber security risk quantification, the focus of this paper, is a vital tool within this broader risk management paradigm, enabling organisations to translate abstract cyber threats into tangible, measurable impacts. A critical component of this proactive management involves advanced cybersecurity monitoring processes, often leveraging tools like Intrusion Detection Systems (IDS) and Intrusion Prevention Systems (IPS) to enhance vigilance and response capabilities [22]. Studies like [23] further emphasise the importance of applying risk analysis to identify threats and countermeasures in specific domains like workstations, which are critical infrastructure components. The pervasive nature of cyber threats means that security and privacy challenges are not unique to financial services but are fundamental considerations across all advanced digital systems, including those in autonomous driving, where comprehensive solutions reviews are continuously needed [24]. Hence, financial services organisations must develop a comprehensive understanding of the multidimensional impacts of potential cyber attacks to mitigate these cyber security risks and ensure the continuity of digital transformation objectives. Failure to do so exposes these organisations to potential financial losses. Oko-Odion et al.’s [25] research backs this point by linking historical financial crises to a lack of understanding of their potential losses. The need for cyber security risk quantification within the financial services industry is widely recognised, with Dupoint [26] opining that it drives financial institutions to invest more heavily in cyber security and cyber resilience. To support this investment decision, Dupoint [26] also acknowledges the lack of a widely accepted approach for systematic assessment of cyber risks’ true impact.

At an elevated view, this practice is evident in retail banks assessing their risks in loan portfolios [27], investment firms diversifying efficiently [28], and insurance companies robustly managing their potential losses [29]. However, a similar outcome-based approach is less mature or completely absent at a lower level within the cyber security domain of financial services. Aggrafiotis et al. [30]’s work backs this position up, advising that the quantification of cyber security impact is still an unsolved problem for organisations. Financial organisations recognise the potential multidimensional impact of cyber security attacks but are unable to translate it into tangible financial terms.

To provide a clear understanding of the obstacles in quantifying cyber risk within this domain, Table 1 summarises the main security challenges and limitations facing digital financial services, including the pervasive issue of data breaches [31].

### 2.2. Taxonomy of the Multidimensional Impact of Cyber Attacks

Quantifying the multidimensional impacts of cyber attacks within digital financial services is dependent on having a comprehensive and agreed-upon impact taxonomy. A digital financial services taxonomy will consistently curate the existing multidimensional impacts, serving as a bedrock for consistent analysis and understanding across stakeholders. Classifying threats, as discussed by Almanasir et al. [33], is a foundational step in establishing such a taxonomy. Within this research area, Greenfield et al. [34], Bouveret [32] and Lis et al. [35], respectively, emphasised the physical, reputational, and economic impacts of cyber attacks. Collectively, these studies highlight the importance of incorporating these three dimensions into a comprehensive digital financial services impact taxonomy.

**Figure 1:**
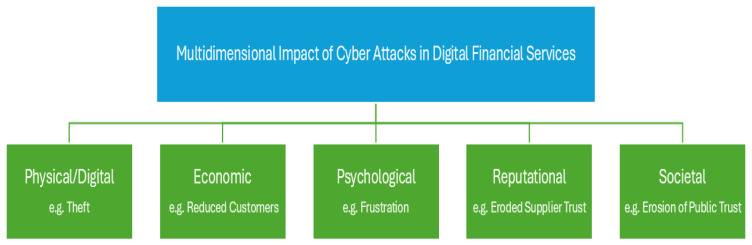
Building blocks of multidimensional taxonomy in digital financial services.

Expanding beyond the context of digital financial services, Agrafiotis et al. [30] emphasised psychological and societal impacts, highlighting their relevance and the need for their inclusion within the scope of digital financial services. By combining these academic perspectives, existing multidimensional impact taxonomy can be refocused for digital financial services. The digital financial taxonomy will cover the full spectrum of the multidimensional impacts, including physical/digital, economic, reputational, psychological, and societal, as shown in Figure 1. It will enable all stakeholders to achieve semantic consistency and shared understanding using precise definitions, standardised metrics and common language. This taxonomy must also include cascading relationships and interdependencies, due to the fact that the impacts of cyber attacks often create ripple effects as a chain reaction, with initial impacts lead to secondary and tertiary consequences. This will further require complex systems models to represent the interdependencies between different impacts and to simulate the propagation effects.

### 2.3. Quantification Techniques Within Digital Financial Services

The implementation of quantification techniques to predict loss is required within insurance [29], investment firms [28], and retail banks, [27]. Expanding beyond the context of financial services includes aviation [36], energy [37], and the manufacturing sector [38]. Combining these academic perspectives reflects that quantification of loss is a well-established practice within financial services leading and expanding into other industries. However, a robust quantification technique for cyber attacks within digital financial services is less mature or completely absent. Aggrafiotis et al. [30]’s work backs this position up, advising that the quantification of cyber security impact is still an unsolved problem for organisations.

The existing quantification techniques used include Value-at-Risk (VaR) [39], QuantTM [40], Conditional Value-at-Risk (CVaR) [41], Real Cyber Value-at-Risk (RCVaR) [42], Business Logic Modelling (BLM) [16], Cyber Value-at-Risk (Cy-VaR), and Breach Level Index (BLI) [43]. While all these techniques have improved the process of finding a solution, they do not cover the full spectrum of the multidimensional impact of cyber attacks. Most of these have either focused on the pre-cyber attack impact or the post-cyber attack impact with huge data inconsistency. Quantifying the multidimensional impacts of cyber attacks within digital financial services is a complex process. It entails translating tangible and intangible losses into quantifiable metrics. Tangible losses, like physical and economic, are easier to quantify as they mostly have direct financial values. Meanwhile, intangible losses like reputational, psychological, and societal losses are subjective, making them more challenging to quantify. Furthermore, effective quantification necessitates the use of robust techniques that can capture the complex and cascading nature of cyber impact.

### 2.4. Application of ML and DL in Cyber Security Risk Quantification

The application of Machine Learning (ML) and Deep Learning (DL) in cyber security has evolved to address the critical need for more accurate and proactive risk quantification, particularly within digital financial services. Rather than focusing solely on threat detection [44], several researchers have applied ML and DL techniques to quantify cyber risks by translating security threats into measurable business impacts. A systematic review by Alshuaibi et al. [45] provides a comprehensive overview of machine learning applications in cybersecurity issues, including risk quantification. For example, advanced machine learning techniques have been successfully applied to enhance DDoS attack detection and mitigation in complex network infrastructures like Software-Defined Networks (SDN) [46] Franco et al. [42] introduced a data-driven framework for cyber risk quantification using a novel Value-at-Risk (VaR) approach; similarly, Kumar et al. [47] demonstrated the use of supervised ML techniques to estimate economic losses from cyber attacks across industries and Orlando et al. [48] also explored ML applications in modelling cyber risk, highlighting how predictive algorithms can be used to simulate future attack scenarios and assess their likely economic consequences. Collectively, these studies provide compelling evidence that ML and DL techniques are not only effective for detection and classification tasks but are also increasingly critical in quantifying cyber security risk. By leveraging predictive modelling, pattern recognition, and probabilistic forecasting, these approaches have helped address limitations in traditional impact modelling, offering scalable and adaptive frameworks for understanding cyber attacks’ financial and operational implications.

## 3. Materials and Methods

This systematic literature review (SLR) aims to identify, analyse, and interpret all available relevant research to quantify the multidimensional impacts of cyber attacks using ML and DL techniques. While it is widely accepted within financial services that a helpful piece of information in deciding investment in cyber security is understanding the potential impact of a cyber attack on that organisation, the complexity due to the multidimensional impacts and evolving threat landscape necessitates an in-depth examination of the current quantification techniques. This SLR investigates the current taxonomy to discuss the multidimensional impacts of cyber attacks, the quantification techniques of cyber attacks with their datasets and the related machine learning implementation use cases, including the algorithms/architecture and their evaluation criteria. Hence, this SLR paper aims to highlight the progress made and the challenges in quantifying the multidimensional impacts of cyber attacks using machine learning.

**Figure 2:**
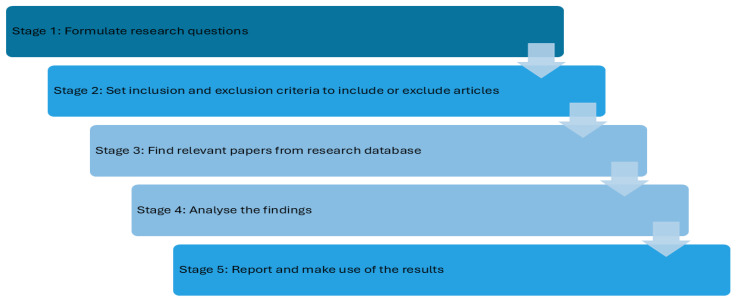
The five stages of the systematic literature review.

The SLR follows the Preferred Reporting Items for Systematic Review (PRISMA) [49] protocol to ensure transparency, reproducibility, and scientific rigour. The five stages observed according to the PRISMA protocols are shown in Figure 2. The stages begin with the formulation of the research questions that focus on the research area. Once the research questions are formulated, a set of inclusion and exclusion criteria is established to focus on relevant and impactful academic articles in line with the research objectives. Following this, relevant academic research articles are identified from the identified databases. Once the data are collected, we analyse the information and discuss the results in the final stage.

Based on this methodology, **44** (out of **637**) articles were chosen, examined, and crticially analysed to ensure a robust review leading to a section on open issues and further research. This allows researchers to research the topic in depth to design a framework for quantifying the multidimensional impact of cyber attacks using machine learning. We have set out to provide detailed coverage of this topic. While this paper aims to comprehensively explore the use of ML and DL techniques to quantify the multidimensional impact of cyber attacks in digital financial services, some limitations exist. Restricting the scope, such as focusing only on English-language publications or specific databases, may lead to the exclusion of relevant studies. Furthermore, publication and selection biases, along with subjective judgments about what constitutes sufficient research, may affect the completeness of this review.

### 3.1. Research Questions

This paper aims to answer the following research questions:

- RQ1: What are the various cyber security impact terminologies used in the literature in the context of digital financial services?
- RQ2: What are the state-of-the-art quantification techniques for the multidimensional impact of cyber security attacks ?
- RQ3: What are the top data sources used by academics and practitioners in quantifying the multidimensional impact of cyber attacks, how are they being used, and what are their limitations?
- RQ4: What are the state-of-the-art implementations of ML and DL in cyber security risk quantification ?

### 3.2. Inclusion and Exclusion Criteria

The inclusion and exclusion criteria have been developed to ensure relevant and valid academic and industry research articles and publications are selected. Thereby enforcing a structured and systematic approach to the literature review.

The inclusion criteria are:

- Scientific and peer-reviewed journals and conference articles
- Relevance to research questions
- Articles relevant to taxonomy, quantifying cyber security impact and ML implementations in cyber security risk quantification
- Articles relevant to digital financial services.
- Articles written in English
- Published any time (year of publication is open and is not limited to a specific period)

The exclusion criteria were:

- Articles that do not discuss the impact of cyber security attacks
- Articles that qualitatively discuss the impact of cyber security attacks without attempting to quantify or measure it
- Unpublished articles, non-peer-reviewed articles, and editorial articles
- Articles that are not fully available through freely published databases and institutional subscriptions
- Non-English articles
- Duplicate publications of the same research

### 3.3. Data Sources

Digital libraries and databases were utilised for comprehensive searches due to their extensive coverage, enhanced search capabilities, accessibility, and convenience. The specific digital libraries used are:

- ACM Digital Library
- Google Scholar
- IEEE Xplore
- MDPI
- PubMed
- ScienceDirect
- SpringerLink

### 3.4. Keywords

A keyword-based search was used to identify relevant academic and industry publications. The keywords were combined using boolean operators such as ‘AND’, ‘OR’, and ‘NOT’.

The main keywords that were utilised are:

- Risk quantification
- Impact quantification
- Taxonomy
- Cyber security impact
- Multidimensional impact
- Financial services
- Machine learning
- Deep learning

### 3.5. Selection of Relevant Articles

The relevant academic and industry publications were selected using the identified keywords. The identified keywords were applied using the inclusion and exclusion criteria on the digital libraries and databases. Three stages were used, as follows:

- **Stage 1—Identification**: The academic articles were identified using a keyword search on the digital libraries and databases. The output of the identification was then filtered based on the inclusion and exclusion criteria.
- **Stage 2—Screening**: The output from the identification stage was then shortlisted based on relevance to the topic and research questions.
- **Stage 3—Eligibility**: The duplicates from the output of the shortlisting stage were then removed.

### 3.6. Data Collection and Items

The data collection process involved a meticulous, paper-by-paper review to identify relevant data to answer the proposed research questions. To ensure objectivity and consistency, this paper followed clear guidelines and a defined process and worked under supervision. A single academic researcher completed the data extraction for this systematic review without using any automation tool. All results compatible with the following data points were collected, covering various measures, time points, and analyses reported within the studies: Outcomes for which data were sought:

- **Author(s)**: Identifying the individuals or entities responsible for the research publication to attribute findings correctly and understand the source.
- **Year of publication**: To track the evolution of cybersecurity impact quantification practices over time and identify trends.
- **Cyber security impact terminology and the number of occurrences**: To capture the specific terminologies used to describe the multidimensional impact of cyber attacks. The number of occurrences was collected for a trend analysis to gauge the prominence and frequency of the terminology usage.
- **Cyber security impact quantification techniques**: This outcome focused on the techniques used to quantify cyber security impact.
- **Commonly used internal and external data sources**: This outcome aimed to identify the types of data sources leveraged for cyber security impact quantification.
- **ML algorithms, DL models implemented and evaluation framework**: This outcome captured instances where machine learning or deep learning techniques were applied to quantify cyber security risk.

### 3.7. Risk of Bias Assessment

This paper did not formally assess the risk of bias within individual academic papers reviewed due to the substantial heterogeneity in study designs, measurement systems, and reported outcomes across the included literature, which rendered the application of standardised bias assessment tools unfeasible. Instead, this paper adopted a qualitative approach during the data extraction process to identify and note significant limitations within each study that could influence the reliability or generalisability of its findings. These limitations primarily included, but were not limited to, the absence of quantitative data for specific outcomes or instances of unclear or inadequately reported methodologies.

### 3.8. Synthesis Methods

This paper developed four syntheses from the four research questions posed earlier on. The four syntheses developed are cyber impact terminologies, cyber impact quantification techniques, data sources and, ML Algorithms and DL models for cyber risk quantification. The mapping of the research questions to these syntheses is shown in Table 2 below.

The eligibility process used to identify academic papers for each specific synthesis is as follows:

- **Cyber impact taxonomy**: This paper applied an additional eligibility layer after the initial inclusion/exclusion screening (as detailed in Section 3.2) for the synthesis focusing on cybersecurity impact terminologies. This process occurred during the comprehensive full-text review of the academic research papers. During this meticulous review, we deemed papers eligible if they mentioned cybersecurity impact terminologies, identifying and coding any stated terms. This layered approach ensured that the final set of selected papers for the cybersecurity impact terminologies synthesis directly addressed this specific outcome, allowing for a comprehensive analysis of the prevalence and context of such language.
- **Cyber impact quantification techniques**: This paper applied an additional eligibility layer after the initial inclusion/exclusion screening (detailed in Section 3.2) for the synthesis focusing on cyber security impact quantification techniques. This process occurred during the comprehensive full-text review. During this meticulous review, we deemed papers eligible if they included cyber security impact quantification techniques. This layered approach ensured that the final set of selected papers for the cyber security impact quantification techniques directly addressed this specific outcome, allowing for a comprehensive analysis of the techniques.
- **Data Sources**: This paper applied an additional eligibility layer after the initial inclusion/exclusion screening (as detailed in Section 3.2) for the synthesis focusing on data sources used in cyber security impact quantification. This process occurred during the comprehensive full-text review. During this meticulous full-text review, we deemed papers eligible if they documented the internal and external data sources used to drive their quantification models. This layered approach ensured that the final set of selected papers identifying the top data sources directly addressed this specific outcome, allowing for a comprehensive analysis of these data sources.
- **ML algorithms and DL models for cyber security risk quantification**: This paper applied an additional eligibility layer after the initial inclusion/exclusion screening (as detailed in Section 3.2) for the synthesis focusing on applications of ML algorithms and DL models in cyber security risk quantification. This process occurred during the comprehensive full-text review. During this meticulous full-text review, we deemed papers eligible if they included the ML algorithms and the DL model implemented and in quantifying cyber security risks. This layered approach ensured that the final set of selected papers with implementations of ML algorithms and DL models for quantifying cyber security risks addressed this specific outcome, allowing for a comprehensive analysis of these implementations.

Likewise, this paper used several methods to tabulate and visually display the results of syntheses, aiming for a clear and comprehensive presentation.

- **Cyber impact taxonomy**: The result of this synthesis is presented in five tables that demonstrate how different cyber impact terminologies are used across five key themes of cyber security impact: physical/digital, economic, psychological, reputational, and societal. These tables consolidate insights from various academic authors. We also created a word cloud to provide a comprehensive view of all cyber security impacts terminologies and their frequency across the included papers.
- **Cyber impact quantification techniques**: The result of this synthesis is presented in a chronological tabular view. This table details academic authors, the specific quantification techniques they employed, and the limitations associated with each technique.
- **Data Sources**: The result of this synthesis includes a tabular view that categorises internal and external data sources. This table also highlights their typical usage and any identified limitations.
- **ML algorithms and DL models for cyber security risk quantification**: The result of this synthesis produces a tabular view that outlines academic authors, relevant use cases, the specific machine learning algorithms applied, and their evaluation criteria for quantifying cyber security risk.

This approach of using various tabular displays and a word cloud allowed us to systematically organise and present complex information from the syntheses. The tables provide structured data that is easy to compare and contrast, while the word cloud offers a visual summary of recurring themes.

### 3.9. Reporting

This paper followed the PRISMA guidelines when conducting this systematic review.

## 4. Analysis of the Results

**Figure 3:**
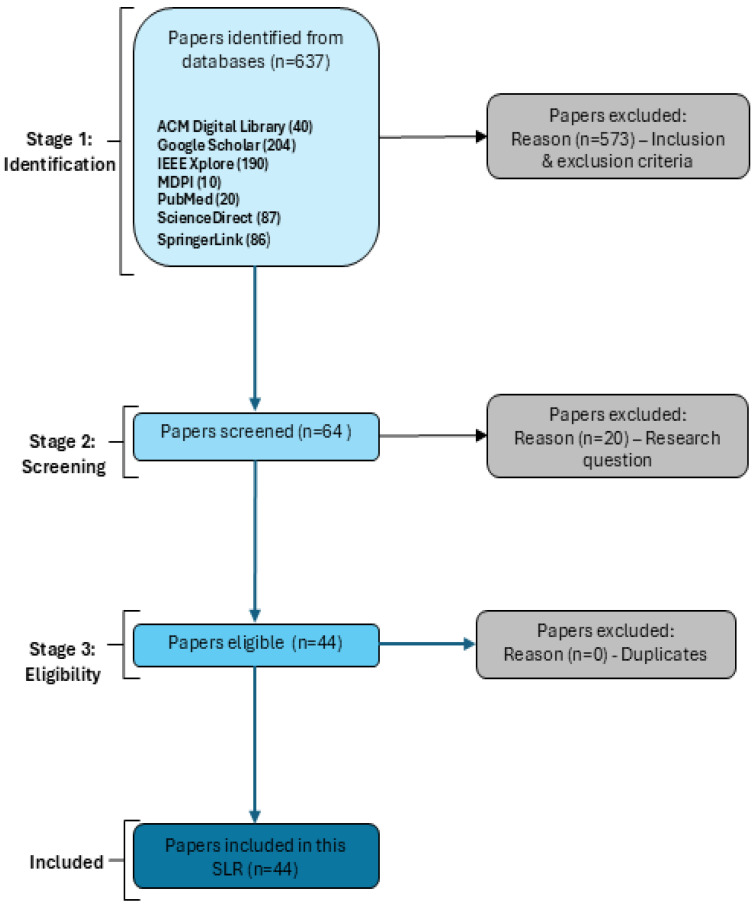
The outcome of the three stage selection process.

The inclusion and exclusion criteria were applied to the collected publications in three stages, according to the PRISMA 2020 protocol [49]. In the first stage, a total of 637 articles were identified from seven different databases: ACM Digital Library (40), Google Scholar (204), IEEE Explore (190), MDPI (10) PubMed (20), ScienceDirect (87), and SpringerLink (86). Afterwards, in stage 2, the collected articles were screened based on the research questions, and the articles that did not meet the inclusion criteria were deemed out of scope and excluded. This resulted in excluding 573 articles and moving forward with 64 articles. Finally, in stage 3, 20 duplicate articles were identified and removed from the 64 articles, leaving 44 articles that were included in this review. The flow diagram of the PRISMA process and the number of articles at each stage is shown in Figure 3.

**Figure 4:**
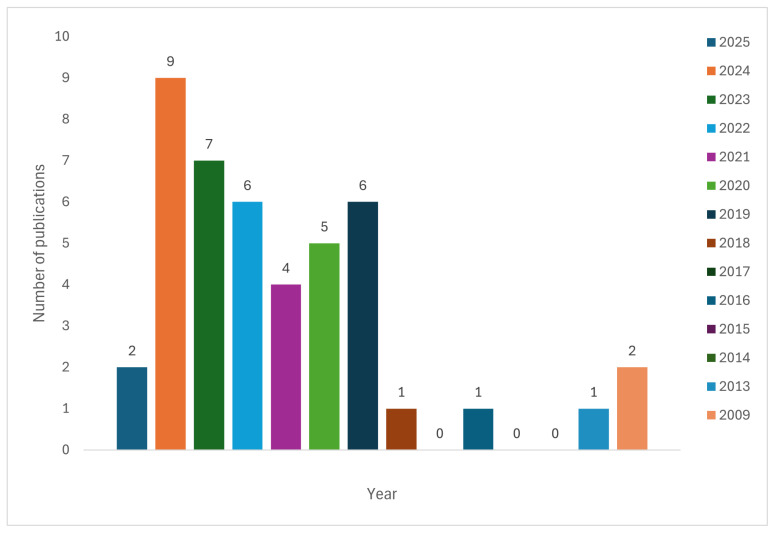
Number of publications per year.

The number of academic articles retrieved from each digital database, along with the number of articles retained after applying the three-stage selection process, is shown in Table 3. The results show that Google Scholar was the richest data source of publications related to quantifying the multidimensional impact of cyber attacks in digital financial services. Also, Figure 4 presents number of publications per year, which can demonstrate a consistent upward trajectory in research productivity, particularly peaking in 2024 with 9 publications, reflecting increased scholarly 3 engagement and output in recent years.

The final publication list that was involved in this review is shown in Table 4, which assigns a publication ID to each publication and includes the authors, year of publication type, and year of publication.

## 5. Results and Discussion

In this section, a synthesis of existing research is presented on the quantification of the multidimensional impact of cyber security attacks. The synthesis details existing research findings investigating the quantification of the multidimensional impact of cyber security attacks. These findings show the key areas of consensus and disagreement within the existing literature and reflect the addressable gaps. The findings also provide a comprehensive overview of the current state-of-the-art in quantifying the multidimensional impact of cyber security attacks.

**RQ1: What are the various cyber security impact terminologies used in academic research papers?**

To address this research question, this paper completed a thematic mapping of cyber impact terminologies. The thematic mapping provided a lens to identify the most frequent, infrequent, and absent terminologies, excluding duplicate terms with identical meanings. The themes used within the thematic mapping are physical/digital, economic, psychological, reputational, and societal (Figure 1). We adopted these themes from Bouveret [32], Greenfield et al. [34], Lis et al. [35], and Agrafiotis et al.’s [30] work due to its robust coverage of all the cyber security impact terminologies. These themes and their associated cyber impact terminologies are as follows:

- ***Physical/Digital Cyber Security Impact Terminology***

This aspect describe the consequences of cyber attacks on material or information technology. We analysed the usage of physical and digital impact terminology across relevant academic research work. Subsequently, we compared the retrieved terms against a comprehensive set of terms gathered from the reviewed research. This mapping revealed the most frequent and infrequent terminology related to physical and digital impacts. The comprehensive set of physical and digital impact terminology used are compromised, infected, corrupted, unavailable, theft, exposed or leaked, bodily injury, loss of life and damage/destruction of equipment. This comprehensive set of terms represents a unique list of core physical and digital cyber security impact terminologies, excluding secondary impacts and synonyms. The analysis of the physical and digital cyber security impact terminologies is presented in Table 5 including citations, and the comprehensive set of physical/digital impact terminologies used across the selected articles.

An analysis of related academic papers reveals that “theft” and “compromised” are the most frequent physical/digital cyber security terms, appearing in 19 and 16 out of 23 research papers, respectively. Following in frequency were “damaged/destruction” (6) and “infected” (5). The least frequent terminologies, each appearing less than 2 across the 23 papers, were “corrupted” [30], “unavailable” [30], “exposed” [30,71], “bodily injury” [30], and “loss of life” [30,35,77]. A plausible reason for this frequency distribution could be because “theft” and “compromised” have established legal definitions as referenced in the Theft Act 1968 [82] and the Computer Misuse Act [83] making them safe words. Other reasons could be researcher bias or lack of a defined domain for physical/digital impacts within the taxonomy of cyber security impacts. As this research has evidenced that no defined domain exists for physical/digital impacts within the taxonomy of cyber security impacts, it should be the most likely cause for this contextual gap. Moreover, terms like “corrupted” [30], “unavailable” [30], “exposed” [30,71], “bodily injury” [30], and “loss of life” [30,35,77], are valid and unique terminologies for describing the physical/digital impacts of cyber security attacks.

- ***Economic Cyber Security Impact Terminology***

This aspect describes the consequences of cyber attacks on financial assets, business operations, and market stability. We analysed the usage of economic impact terminology across relevant academic research work within this research area. Subsequently, this research compared the retrieved terms against a comprehensive set of terms gathered from the reviewed research. This mapping revealed the most frequent and infrequent terminology related to economic impacts. The comprehensive set of economic impact terminology used includes disrupted operations, loss of revenue, reduced customer base, theft or loss of finances/Capital, regulatory fines, extortion payments and a fall in stock price. This comprehensive set of terms represents a unique list of core economics, excluding secondary impacts and synonyms. The analysis of the economic cyber security impact terminologies is presented in Table 6.

An analysis of related academic papers reveals that “loss of revenue” and “reduced customers” are the most frequent economic cyber security terms, appearing in 22 and 20 out of 25 research papers, respectively. Following in frequency were “extortion payments” [16,30,35,55,59] (5), “disrupted operations” [30,39,56,61] (4) and “regulatory fines” [30,34,58] (3). The least frequent terminologies, each appearing once across 25 papers, were “fall in stock price” [30] and “theft or loss of finance capital” [30]. A plausible reason for this frequency distribution could be because “loss of revenue” and “reduced customers” are easier to quantify, widely reported, and often emphasised by board and organisation leaders. Meanwhile, “fall in stock price” or “theft of capital” is delayed/less directly attributable and rarely broken down in breach disclosure reports. Another reason could be a lack of a defined domain for economic impacts within the taxonomy of cyber security impacts. However, for this, both reasons are justifiable later due to the tendency for researchers to naturally gravitate toward the most readily observable and quantifiable metrics in the absence of economic impacts within the taxonomy of cyber security impacts.

- ***Psychological Cyber Security Impact Terminology***

This aspect describes the mental, emotional, and behavioural consequences individuals or organisations experience due to cybe rattacks. This research analysed the usage of psychological impact terminology across relevant academic research work within this research area. Subsequently, this research compared the retrieved terms against a comprehensive set of terms gathered from the reviewed research. This mapping revealed the most frequent and infrequent terminology related to psychological impacts. The comprehensive set of psychological impact terminology used includes confusion, frustration, anxiety/worry, feeling upset, embarrassment, shame, and guilt. This comprehensive set of terms represents a unique list of core psychological, excluding secondary impacts and synonyms. The analysis of the psychological cyber security impact terminologies is presented in Table 7.

An analysis of related academic papers reveals that “worry or anxiety” and “frustration” are the most frequent psychological cyber security terms, appearing in 20 and 18 out of 27 research papers, respectively. Following in frequency were “feeling upset” [30,35,48,55,71,78] (6), “guilty” [16,30,55,58,59] (5), “confusion” [30,39,56,61] (4), and “shame” [16,30,43,78] (4). The least frequent terminology is “embarrassment”, appearing only twice across 27 papers. A plausible reason for this frequency distribution could be that “worry or anxiety” and “frustration” are the initial reactions to cyber security incidents. Other reasons may be due to the focus on organisational or outcomes, and terms like “guilty”, “shame”, and “embarrassment” do come with a potential stigma or vulnerability. The lack of a defined domain for psychological impacts within cyber security taxonomy definitely contributes to this uneven focus, as it hinders comprehensive investigation of the psychological spectrum.

- ***Reputational Cyber Security Impact Terminology***

This aspect describes the consequences on the public image, public perception and trustworthiness of individuals or organisations due to cyber attacks or cyber-related incidents. This research analysed the usage of reputational impact terminology across relevant academic research work within this research area. Subsequently, this research compared the retrieved terms against a comprehensive set of terms gathered from the reviewed research. This mapping revealed the most frequent and infrequent terminology related to psychological impacts. The comprehensive set of psychological impact terminology used includes negative public image, diminished corporate reputation, negative customer sentiment, eroded supplier trust, reduced employer appeal, increased employee turnover, and revocation of credentials. This comprehensive set of terms represents a unique list of core reputational cyber security impacts, excluding secondary impacts and synonyms. The analysis of the reputational cyber security impact terminologies is presented in Table 8.

An analysis of related academic papers reveals that “eroded supplier trust” and “negative customer sentiment” are the most frequent reputational cyber security terms, appearing in 21 and 17 out of 27 research papers, respectively. Following in frequency were “increased employee turnover” [16,30,34,35,55,59] (6), “reduced employer appeal” [30,48,71,77,78] (5), and “negative public image” [30,39,56,61] (4). The least frequent terminologies, each appearing less than thrice across 27 papers, were “revocation of credentials” [30,34,55], and “diminished corporate reputation” [30]. A plausible reason for this frequency distribution could be that “eroded supplier trust” and “negative customer sentiment” are organisation performance metrics primarily included in external financial reports. Meanwhile, “increased employee turnover” and “reduced employer appeal” are included in internal performance reports. The lack of a defined domain for reputational impacts within the cyber security taxonomy contributes to this uneven focus on reputational cyber security impacts.

- ***Societal Cyber Security Impact Terminology***

This aspect describes the consequences of cyber attacks on communities, societies, and even at national levels. This research analysed the usage of societal impact terminology across relevant academic research work within this research area. Subsequently, this research compared the retrieved terms against a comprehensive set of terms gathered from the reviewed research. This mapping revealed the most frequent and infrequent terminology related to psychological impacts. The comprehensive set of societal impact terminology used includes the erosion of public trust, disruption in daily life activities, negative impact on the nation and weakened organisational cohesion. This comprehensive set of terms represents a unique list of core societal cyber security impacts, excluding secondary impacts and synonyms. The analysis of the reputational cyber security impact terminologies is presented in Table 9.

An analysis of related academic papers reveals that “negative impact on nation” and “weakened organisational cohesion” are the most frequent societal cyber security terms, appearing 18 both out of 27 research papers. The least frequent terminologies, each appearing less than 4 across the 27 papers, were “erosion of public trust” [30,39,56,61], and “disruption in daily life activities” [30,40,58]. The frequent appearance of “negative impact on nation” and “weakened organisational cohesion” may be due to their applicability of public used digital services and technology. Conversely, the infrequent terms “erosion of public trust” and “disruption in daily life activities” might be less prioritised in due to a current focus on more immediate operational and national security ramifications. Also, the lack of a clearly defined domain for societal impacts within existing cyber security taxonomies may make societal consequences implicitly discussed within other categories rather than explicitly identified and counted as distinct terms.

**Figure 5:**
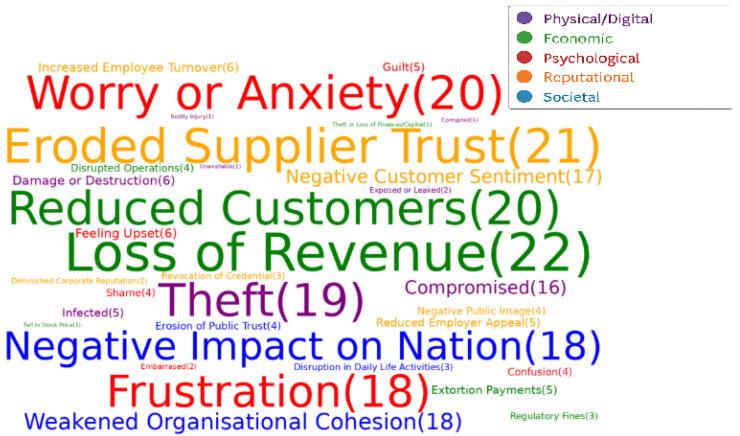
Mutlidimensional cyber attack impact terminologies.

In summary, the various cyber security impact terminologies used in academic research papers are presented in Figure 5. The thematic mapping categorised under physical/digital, economic, psychological, reputational, and societal themes revealed a varied landscape in terminology usage. Notably, terms with established legal definitions (“theft,” “compromised”) tend to be more frequent in the physical/digital domain. At the same time, readily quantifiable metrics (“loss of revenue,” “reduced customers”) dominate the economic discourse. Psychological and reputational impacts see “worry or anxiety”, “frustration”, “eroded supplier trust”, and “negative customer sentiment” as the most frequent terms, respectively, potentially reflecting initial reactions and externally reported metrics. Societal impacts highlight “negative impact on nation” and “weakened organisational cohesion” as prevalent. This research also shows that there are terminology gaps across all the themes of the multidimensional impact of cyber attacks implying gaps in consistent and comprehensive articulation of cyber security impacts within academic literature. The identified lack of clearly defined domains for physical/digital, economic, psychological, reputational, and societal impacts within existing cyber security taxonomies is a likely contributing factor to this uneven distribution and potential contextual gaps. An accepted framework for categorising and describing the multidimensional impacts of cyber attacks will support academic researchers with more defined and quantifiable terms for academic research.

**RQ2: What are the state-of-the-art quantification techniques for the multidimensional impact of cyber security attacks?**

To answer this research question, this paper retrieved and analysed research publications on quantifying the multidimensional impact of cyber security attacks. The results are presented in a chronological order, as shown in Table 10, including the citation, summary of contribution and limitations. The landscape of cyber security impact quantification techniques presents a diverse interplay of methodologies aimed at capturing the multifaceted impacts of cyber attacks. Across the spectrum, these methods can be categorised into pre-attack (PRE) and post-attack (POST) frameworks, each with distinct approaches, strengths, and limitations.

Pre-attack quantification models, such as those in Assen et al. [40], Bentley et al. [56], and Couce-Vieira et al. [59], emphasise proactive assessment, focusing on threat modelling, scenario planning, and business impact analysis. These techniques aim to preemptively estimate potential losses by analysing dependencies [56], business objectives [40], or specific impact categories [59]. The commonality lies in their reliance on structured frameworks, such as CIAA classifications and multivariate models, to map threats to quantifiable impacts. The key contribution or pre-attack quantification is in providing actionable insights to guide resource allocation and prioritise mitigative efforts by integrating threat modelling within business objectives. The emerging need in pre-attack quantification is more automation, scalability, and integration into operational workflows to enhance practical applicability [40,56]. Post-attack quantification models dominate the research landscape, with contributions such as VaR estimation [39,42,48,60], event-study methodologies [58,65,69], and scenario-based case studies [72,78]. These approaches aim to quantify actual damages, including financial losses, reputational harm, and operational impacts. They commonly utilise statistical methods (e.g., copula functions in Bentley et al. [56], Monte Carlo simulations in Thomas et al. [78]) or industry benchmarks in Tahmasebi et al. [77]. The key contribution is that it leverages empirical data to provide accurate cost estimates, which is critical for informing risk mitigation and insurance strategies. The emerging need is that post-attack quantification is hindered due to its data limitations, real-time updates, and holistic impact evaluation, as seen in Dongre et al. [60], and Thomas et al. [78].

The convergence across these research publications is visible in their methodological approach to financial metrics, event-driven insights, and scenario-based approaches. For financial metrics, research papers including Aldasoro et al. [39] (VaR-based calculation), Bentley et al. [56] (copula functions for dependency modelling), and Orlando et al. [48] (Cy-VaR with frequency and severity distributions), emphasise financial quantification. They aim to distill complex impacts into monetary values, often leveraging statistical or actuarial models. For event-driven insights, post-attack models (e.g., Cavusoglu et al. [58], Kamiya et al. [69], and Portela et al. [72]) use event-study methodologies to analyse stock market impacts and immediate financial losses from cyber security breaches. These highlight the real-time repercussions on affected firms and industries. For scenario-based approaches, both pre-cyber attack impact and post-cyber attack impact quantification techniques (e.g., Assen et al. [40]’s threat modelling and Thomas et al. [78]’s Monte Carlo simulation) leverage scenario-driven methods to estimate potential costs and resource implications, enabling a forward-looking perspective or retrospective analysis. The divergence across these research publications is visible in their differentiating techniques across temporary focus, data granularity/sources, methodological depth and impact scope. For temporal focus, pre-attack techniques (e.g., Assen et al. [40]’s QuantTM and Couce-Vieira et al. [59]’s category-specific quantification) prioritise predicting and mitigating risks before incidents occur. These models often incorporate organisational strategy alignment, threat modelling, and resource prioritisation. At the same time, the post-attack techniques (e.g., Franco et al. [42]’s RCVaR and Greenfield et al. [34]’s harm evaluation framework) aim to assess realised impacts, often analysing incident data to refine future risk management strategies. For granularity and sources, Anderson et al. [55] and Tahmasebi et al. [77] rely on industry reports and surveys, offering broad but sometimes shallow insights due to limited datasets, while others, such as Portela et al. [72]’s health sector case study, employ domain-specific and often hypothetical scenarios, which can yield tailored but less generalisable findings. For methodological depth, models such as Bentley et al. [56]’s copula-based multivariate approach explore advanced statistical techniques to capture dependencies and variances, and more straightforward ordinal-based approaches like Facchinetti et al. [63]’s criticality index focus on practical aggregation methods but may lack granularity. For impact scope, Couce-Vieira et al. [59] explores multidimensional impacts, including reputational damage and environmental consequences, and in contrast, Cavusoglu et al. [58] and Goel et al. [65] focus narrowly on financial and stock market repercussions, missing broader socio-economic or operational effects.

The recurring limitations across these research papers underscore key gaps in the field, including standardisation challenges, data/model constraints, integration/accessibility and multidimensional complexity. For standardisation challenges, Aldasoro et al. [39], Eling et al. [62], and Orlando et al. [48] highlight the lack of standardised cyber event definitions, data collection methodologies, and maturity metrics, complicating cross-comparisons and consistency. For data and model constraints, limited datasets (e.g., Anderson et al. [55]) and reliance on assumptions (e.g., Cavusoglu et al. [58], and Kamiya at al., [69]) restrict the accuracy of many models. Expanding data availability and incorporating real-time updates, as noted in Dongre at al., [60], are critical future directions. For integration and accessibility, several techniques, such as Assen et al. [40]’s QuantTM and Couce-Vieira et al.’s category-based modelling, face challenges in integration into organisational workflows and user-friendly applications. Bridging the gap between theoretical models and practical implementation is a pressing need. For multidimensional complexity, Bentley et al. [56], and Orlando et al. [48] tackle dependencies and multidimensional impacts; they simplify key components (e.g., fractional mitigation levels or asset interdependencies). More robust models are needed to capture cascading risks and systemic vulnerabilities.

The insights from these research papers suggest a path forward for cyber impact quantification by integrating pre and post-cyber attack impact quantification approaches, advancing data-driven models, improving standardisation/interoperability, expanding impact dimensions, and a renewed focus on risks and systemic impacts. Integrating pre and post-cyber attack impact quantification approaches will create a hybrid model that combines the foresight of PRE techniques with the precision of POST analyses. For instance, integrating threat modelling [40] with real-world VaR metrics [39], ref. [42] could create robust frameworks for end-to-end risk management. For the advanced data-driven models, enhancing access to anonymised datasets [62] and real-time data updates [60] will be pivotal. As suggested in Franco et al. [42], ML and AI techniques can improve factor calibration and dynamic risk modelling, addressing limitations in current methods. For standardisation and interoperability, establishing universal standards for cyber event definitions, risk metrics, and loss quantification (e.g., Aldasoro et al. [39], Eling et al. [62], and Eling et al. [61]) will facilitate cross-industry benchmarking and the development of globally accepted frameworks. For expanding impact dimensions, Couce-Vieira et al. [59], and Portela et al. [72] highlight the importance of incorporating less tangible impacts into quantification models, such as reputational damage, regulatory penalties, and cascading risks. Tailoring impact prioritisation to organisational profiles will further enhance their relevance. For emerging risks and systemic impacts, Eling et al. [61], and Thomas et al. [78] underscore the need to address systemic risks, including cascading failures and supply chain vulnerabilities. Incorporating agent-based simulation and macro-level risk modelling can address these gaps.

**RQ3: What are the top data sources used by academics and practitioners in quantifying the multidimensional impact of cyber attacks, how are they being used, and what are their limitations?**

To answer this research question, this paper retrieved and analysed the top data sources used in academic research publications on quantifying the multidimensional impact of cyber security attacks. The results are presented in Table 11. The analysis of data sources used in academic research on the multidimensional impact of cyber attacks reveals three prominent intertwined themes: data origin (internal vs. external), temporal applicability (PRE vs. POST attack), and impact type (physical/digital, economic, and reputational). The triangulation of these three themes is required for a complete understanding and empirical quantification of the complex multidimensional impact of cyber attacks.

A holistic computation necessitates the integration of both internal organisational data (e.g., Incident Response Metrics Database, Banking Transactions, Internal Asset Database, Security Control Inventory, and Risk Assessment Reports) and external organisational data (e.g., Operational Riskdata eXchange [84], Privacy Rights Clearinghouse [85], Industry Reports, Advisen Dataset [87], Technology News, and LexisNexis [90]). Internal data is unique to the organisations technnologies, and digital services. However, internal data still needs to be benchmarked with external data to provide a broader context and the necessary industry perspective. However, an apparent difficulty is accommodation and fixing data quality issues. To bring this to life, an organisation seeking to quantify the financial losses from a recent ransomware attack can use its internal data, such as servers and databases from the Internal Asset Database, to identify its crown jewels and vulnerable assets while using external data, such as associated fines from Privacy Rights Clearinghouse [85] and benchmarks data such as the severity of similar cyber attack incidents from the Operational Riskdata eXchange [84] to provide a broader industry perspective.

Crucially, PRE- and POST-attack data are essential for a complete understanding. PRE-attack data, typically sourced from threat models, vulnerability scan reports, and threat intelligence(e.g., Internal Asset Database, Security Control Inventory, Risk Assessment Reports, and potentially Banking Transactions for predictive modelling), facilitates proactive risk assessment and mitigation planning. POST-attack data, derived from incident response logs, financial records, and reputational analysis (e.g., Incident Response Metrics, Operational Riskdata eXchange [84], Privacy Rights Clearinghouse [85], Industry Reports, Advisen Dataset [87], Technology News, and LexisNexis [90]), provides empirical evidence of actual impacts, enabling model calibration and validation. However, PRE-attack data is fundamentally a forward look from the lens of an attacker and includes unverifiable data. On the other hand POST-attack data is based on verifiable data, hence they need to be correctly intertwined to improve the capability to anticipate the multidimensional impact of cyber attacks. To bring this to life, a financial company wanting to understand the reputational impact of a past data breach can model its PRE-attack data, such as emerging threats from its threat intelligence feeds, to model its potential breaches while using POST-attack data, such as articles and reports from Technology and News/Websites to assess the actual reputational damage.

Also, these data sources contribute to quantifying the various dimension of the multidimensional impacts of cyber attacks: physical/digital (e.g., Incident Response Metrics Database), economic (e.g., Operational Riskdata eXchange [84], Privacy Rights Clearinghouse [85], Industry Reports, Advisen Dataset), and reputational (e.g., Technology News/Websites, LexisNexis [90], and potentially survey data—though not explicitly listed, it is often a key component). Integrating data across these three themes—origin, time, and impact—is crucial for comprehensively analysing the multidimensional impact of cyber attacks. To bring this to life, a building society that suffers a cyber attack that disrupts logistics operations and affects customer confidence can understand the multidimensional impact dimensions by analysing physical/digital impact data like downtime of critical systems, the number of affected machines from Incident Response Metrics Database, economic Impact data like average cost of ransomware attacks from Advisen Dataset [87] and reputational impact data like media coverage, public sentiment, and stock price fluctuations post-incident from Technology News/Websites. The company combines data from all three impact categories to understand the attack’s consequences. The Incident Response Metrics Database quantifies the physical/digital disruption, the Advisen Dataset [87] and internal records quantify the economic impact, and media/social media monitoring captures the reputational damage. This comprehensive assessment helps the company quantify the multidimensional impact of cyber attacks to the inform future security investments.

Similarly, these data sources diverge in their practical application, data deficiencies, and Computational intricacies. Internal data sources offer organisation contextual, and nuanced information, that lack external context, whereas external data sources provide industry benchmarks, but may have regionally prejudice or obsolete information.

**RQ4: What are the state-of-the-art implementations of ML and DL in cyber security risk quantification ?**

To answer this question, this paper retrieved and analysed research publications that implement ML and DL models in the quantification of cyber security risks. The results are presented in a chronological order, as shown in Table 12, including the citation, use case, ML/DL algorithms and their evaluation criteria. Table 12 provides a comprehensive overview, detailing how various ML and DL models are specifically applied, their identified use cases, the algorithms employed, and their respective evaluation criteria across the surveyed literature. This systematic presentation provides the foundational context for the subsequent discussion on prevalent algorithmic trends and their nuanced applications within cyber risk quantification.These academic research studies demonstrate a growing integration of ML and DL models for cyber security risk assessment across a wide range of industry sectors. Abdulsatar et al. [50] explored a deep learning-based framework tailored to microservice architectures, leveraging LSTM models to assess risks in highly dynamic and containerised environments. Their work highlights the importance of adaptability and scalability in risk quantification. Similarly, Ahmadi-Assalemi et al. [51] developed a Super Learner Ensemble method for anomaly detection and risk scoring in Industrial Control Systems (ICS), effectively combining multiple ML models to enhance detection accuracy and cyber risk prediction in critical infrastructure. Alagappan et al. [52] focused on the Internet of Things (IoT), employing probabilistic ML techniques to account for the heterogeneity and limited resources typical of IoT devices, emphasising lightweight yet effective quantification models. In the energy domain, Kumar et al. [47] applied deep learning to evaluate the economic impact of cyber threats on Virtual Power Plants (VPPs), illustrating how LSTM models can learn temporal attack patterns and predict potential financial consequences. Alsaadi et al. [54] turned attention to the financial sector, applying predictive analytics through CNNs to model cyber risk trends and support proactive risk mitigation strategies. Also, Yao et al. [81] addressed the construction industry, proposing a machine learning-based framework to assess cyber risks in Building Information Modeling (BIM) systems, underlining the growing relevance of cyber risk management in traditionally non-digital sectors. These studies reveal how ML and DL are increasingly tailored to domain-specific challenges—such as real-time data in ICS, resource constraints in IoT, temporal dependencies in VPPs, and complex data structures in finance and construction. Despite differences in focus, they share a common objective: enhancing cyber security risk quantification by moving beyond basic detection to assess threat likelihood, impact, and severity in contextually rich environments.

Within the analysed body of literature, two distinct trends emerge concerning algorithm selection for cyber security risk quantification: a clear preference for Support Vector Machines (SVM) and Naïve Bayes (NB) among traditional ML techniques, and a comparable emphasis on Convolutional Neural Networks (CNN) and Long Short-Term Memory (LSTM) models within DL approaches. This dual-track adoption highlights how algorithm selection is influenced by data characteristics, computational resources, and the intended application domain. SVM and NB feature prominently across several studies due to their relative interpretability, maturity, and proven effectiveness in classification tasks. SVM, in particular, has been extensively adopted due to its capacity to create optimal decision boundaries (hyperplanes) that separate data into distinct classes, making it well-suited for high-dimensional cyber security datasets. This strength is emphasised in the works of Ahmadi-Assalemi et al. [51], Ali et al. [53], and Biswas et al. [57], who leverage SVM for accurate classification and risk scoring. Further studies by Franco et al. [42] and Rafaiani et al. [73] highlight SVM’s flexibility in handling non-linear data through kernel functions, which transform data into higher-dimensional spaces where linear separability becomes feasible. Such capability is particularly valuable in cyber security, where attack patterns often exhibit complex, non-linear relationships. However, the computational cost associated with training multiple binary classifiers, especially in large or multi-class datasets—poses a scalability challenge. As noted by Franco et al. [64], the One-vs-Rest strategy used by SVM introduces inefficiencies that may hinder real-time applicability in dynamic threat environments.

Contrastingly, NB offers a lightweight, probabilistic alternative that excels in scenarios where rapid inference and uncertainty handling are critical. Studies by Alagappan et al. [52] and Kumar et al. [47] illustrate NB’s utility in modelling probabilistic relationships between features and outcomes, facilitating risk estimation in environments with incomplete or noisy data. NB’s low computational overhead makes it particularly suitable for resource-constrained contexts, such as IoT or edge devices, as supported by findings in Yao et al. [81]. However, its core limitation lies in the assumption of feature independence, which is an unrealistic constraint in many cyber security datasets where features are often correlated. This assumption can compromise accuracy and limit the algorithm’s generalisability in complex threat detection tasks. DL models, specifically LSTMs and CNNs, have gained increasing traction due to their superior capacity for pattern recognition and sequence modelling, both critical for anticipating and quantifying cyber risks in real-time. LSTM models, as adopted by Ali et al. [53], Alsaadi et al. [54], and Goyal et al. [67], are particularly effective in capturing temporal dependencies within time-series cyber data. Their internal architecture, comprising forget, input, and output gates, alongside memory cells, allows LSTMs to preserve long-range dependencies while mitigating vanishing gradient issues common in recurrent neural networks (RNNs). This capability is essential in cyber risk contexts where attack sequences evolve over time and exhibit delayed effects. Studies by Huang et al. [66], Sangiorgio et al. [75], and Yang et al. [80] further confirm LSTM’s ability to detect nuanced patterns and forecast threat trajectories, enhancing both the accuracy and granularity of cyber risk assessments. However, computational intensity and susceptibility to overfitting, especially when training with limited or noisy data, remain key limitations. These factors can hinder the real-time responsiveness and robustness of LSTM-based systems in rapidly changing threat environments.

CNNs, by contrast, offer an alternative DL approach that emphasises spatial feature extraction. As documented by Ali et al. [53] and Alsaadi et al. [54], CNNs utilize convolutional layers to detect hierarchical feature patterns, followed by pooling layers to reduce dimensionality and mitigate overfitting. This architecture enables CNNs to learn abstract representations from raw input data, making them effective in analysing structured inputs such as network traffic matrices, binary code, or log files. In risk quantification contexts, this allows for more refined risk scoring through feature abstraction and anomaly detection. However, as with LSTMs, CNNs are resource-intensive, often requiring large labelled datasets and high-performance computing environments for effective training and deployment. Ali et al. [53] highlight this challenge in their research on efficient training and model scalability, noting that the depth of CNN architectures directly correlates with computational burden and training duration. When comparing ML and DL approaches across the reviewed studies, a consistent pattern emerges: while SVM and NB are favoured for their simplicity, interpretability, and lower computational requirements, LSTM, and CNN models consistently outperform them in terms of predictive accuracy and adaptability to complex data. Ali et al. [53] explicitly note the superior performance of DL models in capturing the intricate, non-linear relationships inherent in cyber security data, which traditional ML models often struggle to represent effectively. However, these performance gains come at a cost. DL models demand substantial training data, are often difficult to interpret, posing challenges for explainability in high-stakes environments, and are prone to overfitting, particularly when domain-specific data is scarce.

In summary, the reviewed literature reflects a progressive evolution from traditional ML to more sophisticated DL techniques in cyber risk quantification, driven by the need for models that can effectively process high-volume, high-velocity, and high-variety data. While ML algorithms like SVM and NB continue to offer valuable trade-offs between accuracy and efficiency in constrained environments, DL models such as LSTM and CNN are becoming increasingly essential for developing predictive, adaptive, and context-aware risk assessment frameworks. The selection of algorithms, therefore, hinges on a careful balancing of performance needs, computational capacity, data availability, and the specific threat landscape of the application domain.

## 6. Open Issues and Future Research Directions

The deployment of digital human-centric services by financial organisations continues to increase their cyber attack surface. Consequently, a proactive approach to cyber security is a must. Organisations need to be able to quantify the multidimensional impact of cyber attacks before they occur. This capability is now a hygiene factor in information and cyber security. Quantifying the multidimensional impact of cyber attacks before they occur will help organisations subscribe to the right risk tolerance and manage their cyber security budget. This quantification also facilitates the creation of a healthy balance between securing digital services and providing consumers with the necessary business functionalities. Ultimately, this will allow organisations to deploy appropriate risk-based controls. There are significant gaps in the existing academic and industry body of knowledge required to deliver this capability. The significant gaps and future research directions include the following:

### 6.1. Standardisation of Digital Financial Services Taxonomy

One of the key challenges in assessing the impact of cyber attacks on digital financial services is the lack of a standardised taxonomy for categorising and describing their multidimensional effects. This inconsistency hinders effective communication, comparison across studies, and the development of robust analytical models. To address this, it is essential to develop and promote the adoption of a comprehensive, universally accepted digital financial taxonomy. This taxonomy should feature clearly defined domains (physical/digital, economic, psychological, reputational, societal) and standardised terminology to ensure consistent and nuanced descriptions of the multidimensional impact of cyber attacks in academic research and practice. A standardised taxonomy will reduce ambiguity, support comparative studies, and enable more effective quantification of cyber attack impact. Furthermore, curated libraries of the multidimensional cyber impact terms, use cases, and examples, should be developed and shared to aid researchers in selecting appropriate, standardised language for their work and reduce duplication.

### 6.2. Interdisciplinary Approaches for Multidimensional Impact

A key challenge in understanding the full scope of cyber attack impacts lies in the fragmented and discipline-specific approaches currently adopted. This often results in a narrow view that fails to capture the complex, interrelated consequences of such attacks on digital financial services and broader society. To overcome these limitations, future research should adopt a truly interdisciplinary approach. Drawing on insights from psychology, sociology, law, economics, and other relevant fields will enable researchers to examine cyber attacks from multiple dimensions—emotional, social, legal, and financial. This integration of diverse perspectives will not only enhance the granularity and depth of analysis but also support the development of more effective response strategies, policies, and resilience frameworks that reflect the real-world complexity of cyber threats.

### 6.3. Real-Time Impact Quantification

One of the most pressing gaps in the field of cyber impact quantification is the lack of real-time or near-real-time assessment frameworks. Existing models are largely retrospective, focusing on post-attack evaluations based on historical data or simulated scenarios. However, in high-risk sectors such as digital financial services, the ability to quantify impact dynamically as an attack unfolds is crucial for mitigating loss and informing timely decision-making. Real-time quantification would enable organisations to triage responses based on the evolving severity and scope of an attack, support more effective communication with stakeholders and regulators, and facilitate rapid financial and operational adjustments. Achieving this requires integrating high-frequency telemetry data, real-time threat intelligence feeds, and adaptive ML/DL models capable of updating predictions as new data becomes available. Furthermore, these systems must be resilient to noise and incomplete information, particularly in the early stages of an incident. Research should also explore the role of digital twins or simulation-driven forecasting within live environments to estimate likely downstream effects. Developing such real-time capabilities presents not only technical but also organisational and regulatory challenges, such as ensuring data reliability, minimising latency, and aligning with incident response protocols. Nevertheless, real-time impact quantification represents a critical frontier for enhancing cyber resilience and operational agility.

### 6.4. Hybrid Quantification Framework for Cyber Impact

A major limitation in current cyber risk assessment is the absence of a unified framework capable of capturing the full spectrum of a cyber attack’s multidimensional impact. Existing models often focus solely on either pre-attack predictions or post-attack consequences, resulting in fragmented and less actionable insights. To address this, a hybrid quantification framework is needed—one that seamlessly integrates both pre- and post-attack dimensions. This includes combining predictive tools such as threat modelling and scenario planning with post-attack analytical methods like value-at-risk (VaR) estimation and event studies. By merging proactive foresight with reactive precision, the framework would enable a more comprehensive and dynamic understanding of cyber attack impacts, supporting stronger end-to-end risk management. Additionally, the quantification techniques should be tailored to reflect sector-specific characteristics and regional contexts. Customised models that incorporate unique threat vectors, regulatory requirements, and operational interdependencies will yield more precise, relevant, and actionable outcomes.

### 6.5. Enhancing Data Utilisation in Impact Quantification

A key challenge in accurately quantifying the multidimensional impact of cyber attacks is the underutilisation and fragmentation of available data. Current approaches often lack the integration of diverse data sources and fail to contextualise information within specific organisational environments. To address this, a more strategic and holistic approach to data utilisation is essential. This includes combining internal, organisation-specific data with broader external benchmarks, and aligning pre-attack predictive insights with post-attack empirical evidence. Seamless integration across impact dimensions, such as physical/digital, economic, and reputational, is also critical. Addressing data quality and accessibility issues through improved data governance, as well as exploring novel and unconventional data sources, will further strengthen this effort. Ultimately, developing methodologies that contextualise external data within the unique operational landscape of an organisation will enable more accurate, relevant, and actionable impact assessments, contributing to enhanced cyber resilience and informed decision-making.

### 6.6. Integrating ML/DL into Quantification Frameworks

A significant barrier to progress in cyber impact quantification is the lack of intelligent, adaptive models capable of handling the complexity and variability of cyber threats. To overcome this, integrating ML and DL into quantification frameworks is essential. These technologies can unlock new capabilities by analysing large-scale, multidimensional data from both internal and external sources, and by combining pre-attack predictions with post-attack impact assessments. Key priorities should include the development of hybrid models, ensuring explainability and interpretability, and tailoring solutions to specific sectors and use cases. Addressing data quality, volume, and temporal dynamics, through sequential data analysis, is critical for effective model training. Moreover, performance should be evaluated using comprehensive, multidimensional metrics that reflect real-world complexities. Ultimately, contextualising external data within unique organisational environments through ML/DL models will yield more accurate, actionable insights, enhancing both organisational resilience and broader societal cyber security.

## 7. Conclusions

Quantifying the multidimensional impacts of cyber attacks remains a significant challenge for all organisations across various sectors, including those within digital financial services. However, there are promising approaches to creating a robust solution. Firstly, curating a universally accepted and comprehensive impact taxonomy is crucial across industries to serve as a bedrock for consistent analysis and understanding across stakeholders. Secondly, enhancing the availability and quality of both internal organisational data and external data will baseline and improve the accuracy and robustness of quantification models for any entity facing cyber risks. Finally, implementing ML algorithms and DL models offers quantification techniques that integrate pre-attack threats with post-attack consequences, leading to a complete calculation of the multidimensional cyber security impacts in diverse contexts. This paper presented a comprehensive systematic review with a thematic assessment of terminologies for quantifying cyber attack impact. Specifically, this review examines the terminologies used to describe these impacts, the quantification techniques employed, the available internal and external data sources, and the applications of ML and DL in cyber security risk quantification. Following a rigorous search strategy, 44 articles were selected from an initial pool of 637 publications across multiple reputable databases. These selected articles were examined to evaluate recent advancements in quantifying the multidimensional impact of cyber attacks. Consequently, this paper identifies common impact terminologies and quantification techniques, frequently used datasets, and the implementation of ML and DL in cyber security risk quantification. Lastly, this paper presents critical open issues and research directions essential for future progress in this research area. These include standardisation of cyber impact taxonomy across different domains, fostering interdisciplinary approaches to improve analysis of impact terminology across research disciplines, ML- and DL-enhanced quantification frameworks, and enhancing data utilisation. This systematic review, by consolidating current knowledge and identifying critical gaps, provides the essential foundation for future works to propose a refined taxonomy and develop robust, practical frameworks for cyber risk quantification that can be applied more broadly. Implementing these recommendations is essential for creating a comprehensive and practical framework for quantifying the multidimensional impact of cyber attacks.

## Figures and Tables

**Table 1:** Main Security Challenges and Limitations in Digital Financial Services.

Challenge/Limitation	Description/Impact
Lack of Standardised Impact Taxonomy [30]	Difficulty in consistently defining, categorising, and communicating the full spectrum of physical, digital, economic, psychological, reputational, and societal impacts, leading to inconsistent measurement and understanding.
Complexity of Multidimensional Impacts [14]	Cyber attacks trigger a cascade of interconnected effects beyond direct financial loss, including intangible consequences (e.g., trust erosion, reputational damage, psychological distress), which are inherently difficult to quantify.
Absence of Mature Quantification Models [16,30]	Unlike other financial risks (e.g., credit risk), there is a lack of widely accepted, robust, and comprehensive quantification methodologies specifically tailored to the unique complexities of cyber risks in DFS.
Data Scarcity and Inconsistency	Limited availability of high-quality, standardised, and granular data on past cyber incidents and their full multidimensional impacts, hindering the development and validation of accurate quantification models.
Evolving Threat Landscape [7,8]	The dynamic nature of cyber threats, attack vectors, and attacker motivations constantly shifts, making it challenging to develop static quantification models that remain relevant and predictive over time.
Legacy Infrastructure [32]	Many institutions operate on outdated or hybrid IT systems, introducing vulnerabilities.
Human Error and Insider Threats [8]	Mistakes by employees and contractors remain a persistent risk.

**Table 2:** Mapping of research questions to synthesis topics.

ID	Research Question(s)	Synethesis Topic(s)
1	What are the various cyber security impact terminologies used in the literature in the context of digital financial services?	Cyber impact taxonomy
2	What are the state-of-the-art quantification techniques for the multidimensional impact of cyber security attacks?	Cyber impact quantification techniques
3	What are the top data sources used by academics and practitioners in quantifying the multidimensional impact of cyber attacks, how are they being used, and what are their limitations?	Data Sources
4	What are the state-of-the-art implementations of ML and DL in cyber security risk quantification?	ML algorithms and DL models for cyber risk quantification

**Table 3:** The number of search result per database after applying three stages of the selection process.

Database	Stage 1	Stage 2	Stage 3
ACM Digital Library	40	8	4
Google Scholar	204	20	17
IEEE Explore	190	9	7
MDPI	10	5	2
PubMed	20	3	1
ScienceDirect	87	9	6
SpringerLink	86	10	7
**Total**	**637**	**64**	**44**

**Table 4:** Retrieved publications that are related to research questions.

ID	Citation	Publication Type	Year
1	Abdulsatar et al. [50]	Journal	2024
2	Ahmadi-Assalemi et al. [51]	Journal	2022
3	Agrafiotis et al. [30]	Journal	2018
4	Alagappan et al. [52]	Journal	2022
5	Aldasoro et al. [39]	Journal	2020
6	Ali et al. [53]	Journal	2025
7	Alsaadi et al. [54]	Journal	2024
8	Anderson et al. [55]	Conference	2019
9	Assen et al. [40]	Journal	2024
10	Bentley et al. [56]	Journal	2020
11	Biswas et al. [57]	Journal	2024
12	Bouveret [32]	Journal	2019
13	Cavusoglu et al. [58]	Journal	2024
14	Couce-Vieira et al. [59]	Journal	2020
15	Dongre et al. [60]	Book Chapter	2019
16	Eling et al. [61]	Journal	2023
17	Eling et al. [62]	Journal	2016
18	Erola et al. [41]	Journal	2022
19	Facchinetti et al. [63]	Journal	2020
20	Franco et al. [42]	Journal	2024
21	Franco et al. [64]	Journal	2022
22	Goel et al. [65]	Journal	2009
23	Huang et al. [66]	Journal	2022
24	Goyal et al. [67]	Journal	2009
25	Greenfield et al. [34]	Conference	2022
26	Kalinin et al. [68]	Journal	2021
27	Kamiya et al. [69]	Journal	2021
28	Kumar et al. [47]	Journal	2021
29	Lazrag et al. [16]	Book Chapter	2025
30	Lis et al. [35]	Journal	2019
31	Orlando [48]	Journal	2021
32	Pala et al. [70]	Journal	2019
33	Pollmeier et al. [71]	Journal	2023
34	Portela et al. [72]	Journal	2023
35	Rafaiani et al. [73]	Journal	2023
36	Razavi et al. [74]	Journal	2023
37	Sangiorgio et al. [75]	Journal	2020
38	Shevchenko et al. [76]	Journal	2023
39	Tahmasebi et al. [77]	Journal	2024
40	Thomas et al. [78]	Journal	2013
41	Wickramasinghe Anuja [79]	Conference	2024
42	Yang et al. [80]	Journal	2019
43	Yao et al. [81]	Journal	2024
44	Zadeh et al. [43]	Journal	2023

**Table 5:** Physical/digital impact terminologies.

Citation	Compromised	Infected	Corrupted	Unavailable	Theft	Exposed or Leaked	Bodily Injury	Loss of Life	Damage or Destruction
Agrafiotis et al. [30]	**✓**	**✓**	**✓**	**✓**	**✓**	**✓**	**✓**	**✓**	**✓**
Eling et al. [61]	**✓**	**X**	**X**	**X**	**✓**	**X**	**X**	**X**	**✓**
Aldasoro et al. [39]	**X**	**X**	**X**	**X**	**✓**	**X**	**X**	**X**	**✓**
Bentley et al. [56]	**X**	**X**	**X**	**X**	**✓**	**X**	**X**	**X**	**✓**
Assen et al. [40]	**✓**	**X**	**X**	**X**	**X**	**X**	**X**	**X**	**✓**
Thomas et al. [78]	**✓**	**✓**	**X**	**X**	**✓**	**X**	**X**	**X**	**X**
Greenfield et al. [34]	**X**	**X**	**X**	**X**	**✓**	**X**	**X**	**X**	**X**
Anderson et al. [55]	**✓**	**✓**	**X**	**X**	**✓**	**X**	**X**	**X**	**X**
Kimaya et al. [69]	**X**	**X**	**X**	**X**	**✓**	**X**	**X**	**X**	**X**
Eling et al. [62]	**✓**	**X**	**X**	**X**	**✓**	**X**	**X**	**X**	**X**
Goel et al. [65]	**✓**	**X**	**X**	**X**	**✓**	**X**	**X**	**X**	**X**
Cavusoglu et al. [58]	**X**	**X**	**X**	**X**	**✓**	**X**	**X**	**X**	**✓**
Dongre et al. [60]	**✓**	**X**	**X**	**X**	**✓**	**X**	**X**	**X**	**X**
Lis et al. [35]	**X**	**✓**	**X**	**X**	**✓**	**X**	**X**	**✓**	**X**
Bouveret et al. [32]	**✓**	**X**	**X**	**X**	**✓**	**X**	**X**	**X**	**X**
Shevchenko et al. [76]	**✓**	**X**	**X**	**X**	**✓**	**X**	**X**	**X**	**X**
Tahmasebi et al. [77]	**✓**	**X**	**X**	**X**	**✓**	**X**	**X**	**✓**	**X**
Erola et al. [41]	**✓**	**X**	**X**	**X**	**✓**	**X**	**X**	**X**	**X**
Zadeh et al. [43]	**✓**	**X**	**X**	**X**	**✓**	**X**	**X**	**X**	**X**
Pollmeier et al. [71]	**✓**	**✓**	**X**	**X**	**X**	**✓**	**X**	**X**	**X**
Portela et al. [72]	**✓**	**X**	**X**	**X**	**X**	**X**	**X**	**X**	**X**
Razavi et al. [74]	**✓**	**X**	**X**	**X**	**X**	**X**	**X**	**X**	**X**
Couce-Vieira et al. [59]	**X**	**X**	**X**	**X**	**✓**	**X**	**X**	**X**	**X**

**Table 6:** Economic impact terminologies.

Citation	Disrupted Operations	Loss of Revenue	Reduced Customers	Fall in Stock Price	Theft or Loss of Finances/Capital	Regulatory Fines	Extortion Payments
Agrafiotis et al. [30]	**✓**	**✓**	**✓**	**✓**	**✓**	**✓**	**✓**
Eling et al. [61]	**✓**	**✓**	**✓**	**X**	**X**	**X**	**X**
Aldasoro et al. [39]	**✓**	**X**	**✓**	**X**	**X**	**X**	**X**
Bentley et al. [56]	**✓**	**✓**	**✓**	**X**	**X**	**X**	**X**
Assen et al. [40]	**X**	**✓**	**X**	**X**	**X**	**X**	**X**
Thomas et al. [78]	**X**	**✓**	**✓**	**X**	**X**	**X**	**X**
Greenfield et al. [34]	**X**	**X**	**✓**	**X**	**X**	**✓**	**X**
Anderson et al. [55]	**X**	**✓**	**✓**	**X**	**X**	**X**	**✓**
Kimaya et al. [69]	**X**	**X**	**✓**	**X**	**X**	**X**	**X**
Eling et al. [62]	**X**	**✓**	**✓**	**X**	**X**	**X**	**X**
Goel et al. [65]	**X**	**✓**	**✓**	**X**	**X**	**X**	**X**
Cavusoglu et al. [58]	**X**	**✓**	**✓**	**X**	**X**	**✓**	**X**
Dongre et al. [60]	**X**	**✓**	**✓**	**X**	**X**	**X**	**X**
Lis et al. [35]	**X**	**✓**	**✓**	**X**	**X**	**X**	**✓**
Bouveret et al. [32]	**X**	**✓**	**✓**	**X**	**X**	**X**	**X**
Shevchenko et al. [76]	**X**	**✓**	**✓**	**X**	**X**	**X**	**X**
Tahmasebi et al. [77]	**X**	**✓**	**✓**	**X**	**X**	**X**	**X**
Erola et al. [41]	**X**	**✓**	**✓**	**X**	**X**	**X**	**X**
Zadeh et al. [43]	**X**	**✓**	**✓**	**X**	**X**	**X**	**X**
Pollmeier et al. [71]	**X**	**✓**	**X**	**X**	**X**	**X**	**X**
Portela et al. [72],	**X**	**✓**	**X**	**X**	**X**	**X**	**X**
Razavi et al. [74]	**X**	**✓**	**X**	**X**	**X**	**X**	**X**
Couce-Vieira et al. [59]	**X**	**X**	**X**	**X**	**X**	**X**	**✓**
Orlando et al. [48]	**X**	**✓**	**X**	**X**	**X**	**X**	**X**
Franco et al. [42]	**X**	**✓**	**✓**	**X**	**X**	**X**	**X**
Kumar et al. [47]	**X**	**✓**	**✓**	**X**	**X**	**X**	**X**
Lazrag et al. [16]	**X**	**X**	**X**	**X**	**X**	**X**	**✓**

**Table 7:** Psychological impact terminologies.

Citation	Confusion	Frustration	Worry or Anxiety	Feeling Upset	Embarrassed	Shame	Guilt
Agrafiotis et al. [30]	**✓**	**✓**	**✓**	**✓**	**✓**	**✓**	**✓**
Eling et al. [61]	**✓**	**✓**	**✓**	**X**	**X**	**X**	**X**
Aldasoro et al. [39]	**✓**	**✓**	**X**	**X**	**X**	**X**	**X**
Bentley et al. [56]	**✓**	**✓**	**✓**	**X**	**X**	**X**	**X**
Assen et al. [40]	**X**	**X**	**✓**	**X**	**X**	**X**	**X**
Thomas et al. [78]	**X**	**✓**	**✓**	**✓**	**X**	**✓**	**X**
Greenfield et al. [34]	**X**	**✓**	**X**	**X**	**X**	**X**	**X**
Anderson et al. [55]	**X**	**✓**	**✓**	**✓**	**X**	**X**	**✓**
Kimaya et al. [69]	**X**	**✓**	**X**	**X**	**X**	**X**	**X**
Eling et al. [62]	**X**	**✓**	**✓**	**X**	**X**	**X**	**X**
Goel et al. [65]	**X**	**✓**	**✓**	**X**	**X**	**X**	**X**
Cavusoglu et al. [58]	**X**	**✓**	**X**	**X**	**X**	**X**	**✓**
Dongre et al. [60]	**X**	**✓**	**✓**	**X**	**X**	**X**	**X**
Lis et al. [35]	**X**	**✓**	**X**	**✓**	**✓**	**X**	**X**
Bouveret et al. [32]	**X**	**✓**	**✓**	**X**	**X**	**X**	**X**
Shevchenko et al. [76]	**X**	**✓**	**✓**	**X**	**X**	**X**	**X**
Tahmasebi et al. [77]	**X**	**✓**	**✓**	**X**	**X**	**X**	**X**
Erola et al. [41]	**X**	**✓**	**✓**	**X**	**X**	**X**	**X**
Zadeh et al. [43]	**X**	**✓**	**✓**	**X**	**X**	**✓**	**X**
Pollmeier et al. [71]	**X**	**X**	**✓**	**✓**	**X**	**X**	**X**
Portela et al. [72],	**X**	**X**	**✓**	**X**	**X**	**X**	**X**
Razavi et al. [74]	**X**	**X**	**✓**	**X**	**X**	**X**	**X**
Couce-Vieira et al. [59]	**X**	**X**	**X**	**X**	**X**	**X**	**✓**
Orlando et al. [48]	**X**	**X**	**✓**	**✓**	**X**	**X**	**X**
Franco et al. [42]	**X**	**X**	**✓**	**X**	**X**	**X**	**X**
Kumar et al. [47]	**X**	**X**	**✓**	**X**	**X**	**X**	**X**
Lazrag et al. [16]	**X**	**X**	**X**	**X**	**X**	**✓**	**✓**

**Table 8:** Reputational impact terminologies.

Citation	Negative Public Image	Diminished Corporate Reputation	Negative Customer Sentiment	Eroded Supplier Trust	Reduced Employer Appeal	Increased Employee Turnover	Revocation of Credentials
Agrafiotis et al. [30]	**✓**	**✓**	**✓**	**✓**	**✓**	**✓**	**✓**
Eling et al. [61]	**✓**	**X**	**✓**	**✓**	**X**	**X**	**X**
Aldasoro et al. [39]	**✓**	**X**	**✓**	**X**	**X**	**X**	**X**
Bentley et al. [56]	**✓**	**X**	**✓**	**✓**	**X**	**X**	**X**
Assen et al. [40]	**X**	**X**	**X**	**✓**	**X**	**X**	**X**
Thomas et al. [78]	**X**	**X**	**✓**	**✓**	**✓**	**X**	**X**
Greenfield et al. [34]	**X**	**X**	**✓**	**X**	**X**	**✓**	**✓**
Anderson et al. [55]	**X**	**X**	**✓**	**✓**	**X**	**✓**	**✓**
Kimaya et al. [69]	**X**	**X**	**✓**	**X**	**X**	**X**	**X**
Eling et al. [62]	**X**	**X**	**✓**	**✓**	**X**	**X**	**X**
Goel et al. [65]	**X**	**X**	**✓**	**✓**	**X**	**X**	**X**
Cavusoglu et al. [58]	**X**	**✓**	**✓**	**✓**	**X**	**X**	**X**
Dongre et al. [60]	**X**	**X**	**✓**	**✓**	**X**	**X**	**X**
Lis et al. [35]	**X**	**X**	**✓**	**X**	**X**	**✓**	**X**
Bouveret et al. [32]	**X**	**X**	**✓**	**✓**	**X**	**X**	**X**
Shevchenko et al. [76]	**X**	**X**	**✓**	**✓**	**X**	**X**	**X**
Tahmasebi et al. [77]	**X**	**X**	**✓**	**✓**	**✓**	**X**	**X**
Erola et al. [41]	**X**	**X**	**✓**	**✓**	**X**	**X**	**X**
Zadeh et al. [43]	**X**	**X**	**✓**	**✓**	**X**	**X**	**X**
Pollmeier et al. [71]	**X**	**X**	**X**	**✓**	**✓**	**X**	**X**
Portela et al. [72]	**X**	**X**	**X**	**✓**	**X**	**X**	**X**
Razavi et al. [74]	**X**	**X**	**X**	**✓**	**X**	**X**	**X**
Couce-Vieira et al. [59]	**X**	**X**	**X**	**X**	**X**	**✓**	**X**
Orlando et al. [48]	**X**	**X**	**X**	**✓**	**✓**	**X**	**X**
Franco et al. [42]	**X**	**X**	**X**	**✓**	**X**	**X**	**X**
Kumar et al. [47]	**X**	**X**	**X**	**✓**	**X**	**X**	**X**
Lazrag et al. [16]	**X**	**X**	**X**	**X**	**X**	**✓**	**X**

**Table 9:** Societal impact terminologies.

Citation	Erosion of Public Trust	Disruption in Daily Life Activities	Negative Impact on Nation	Weakened Organisational Cohesion
Agrafiotis et al. [30]	**✓**	**✓**	**✓**	**✓**
Eling et al. [61]	**✓**	X	**✓**	**✓**
Aldasoro et al. [39]	**✓**	X	**✓**	X
Bentley et al. [56]	**✓**	X	**✓**	**✓**
Assen et al. [40]	X	**✓**	X	**✓**
Thomas et al. [78]	X	X	**✓**	**X**
Greenfield et al. [34]	X	X	**✓**	X
Anderson et al. [55]	X	X	**✓**	**✓**
Kimaya et al. [69]	X	X	**✓**	X
Eling et al. [62]	X	X	**✓**	X
Goel et al. [65]	X	X	**✓**	**✓**
Cavusoglu et al. [58]	X	**✓**	**✓**	X
Dongre et al. [60]	X	X	**✓**	**✓**
Lis et al. [35]	X	X	**✓**	X
Bouveret et al. [32]	X	X	**✓**	**✓**
Shevchenko et al. [76]	X	X	**✓**	**✓**
Tahmasebi et al. [77]	X	X	**✓**	**✓**
Erola et al. [41]	X	X	**✓**	**✓**
Zadeh et al. [43]	X	X	**✓**	**✓**
Pollmeier et al. [71]	X	X	X	**✓**
Portela et al. [72]	X	X	X	**✓**
Razavi et al. [74]	X	X	X	**✓**
Couce-Vieira et al. [59]	X	X	X	X
Orlando et al. [48]	X	X	X	**✓**
Franco et al. [42]	X	X	X	**✓**
Kumar et al. [47]	X	X	X	**✓**
Lazrag et al. [16]	X	X	X	X

**Table 10:** Quantification techniques and limitations.

Citation	Quantification Technique	Limitations
Aldasoro et al. [39]	Post-attack impact quantification method which estimates cyber event losses as a subset of operational losses using Value-at-Risk (VaR).	The lack of a standardised definition of cyber events leads to inconsistent data interpretation/risk classification and reduced Value-at-Risk (VaR) reliability.
Anderson et al. [55]	Post-attack impact quantification from surveys filtering by cyber crime types and their associated costs.	There is limited reference data in surveys to calculate the cost of cyber crimes.
Assen et al. [40]	Pre-attack impact quantification by building quantitative models to measure the economic impact of identified threats and calculating potential financial losses and other associated costs.	This technique has not been evaluated across diverse organisational contexts, lacks strategies to integrate into development processes, and the current implementation is manual.
Bentley et al. [56]	Pre-attack impact quantification using a multivariate model constructed from a copula function and marginal distributions with the representation of multiple damages with varying frequencies and impact sizes.	This technique has not been evaluated on advanced risk measures, standard shock models, and discrete mitigation modelling techniques and inherits the limitations of VaR.
Bouveret et al. [32]	Post-attack impact quantification technique using either historical, variance-covariance, or Monte Carlo VaR calculation methods. The input includes scope, time horizon, and confidence level for estimating loss distribution parameters.	This technique depends on data from the ORX [84] database, which does not have a standardised definition of cyber events, leading to inconsistent data interpretation/risk classification and reduced Value-at-Risk (VaR) reliability
Cavusoglu et al. [58]	Post-attack impact quantification technique using event-study analysis, including the direct financial cost and information transfer of cyber security breaches in affected firms and security technology providers.	The event-study analysis depends on the assumption of an efficient market and rational investors, which does not hold. Hence, economic analysis requires additional normalisation.
Couce-Vieira et al. [59]	Pre-attack impact quantification technique of quantifying various impact categories using specific methodologies involving modelling, parameter estimation (often with expert judgment), data incorporation, and Bayesian updating to arrive at a quantified impact value.	The lack of tailored impact prioritisation profiles, the absence of formalised attack trees/types, and the non-incorporation of uncertainty concerning attacker preferences through random utility functions.
Dongre et al. [60]	Post-attack impact quantification using a mathematical formula that expresses the cost impacts of data breaches using cost components.	This technique does not consider the importance of operational impact, dynamic downtime, and business function.
Eling et al. [62]	Post-attack impact quantification framework using the cost per incident and cost per record of a data breach.	There is a deficiency in anonymised data for analysis of extreme loss scenarios.
Eling et al. [61]	Post-attack impact quantification technique, including a unified language, scenario, and sensitivity analysis for describing cyber scenarios, then using the input–output model to model the economic impact of cyber risk scenarios.	The technique does not factor in all cyber attack scenarios, including emerging threats and novel attack vectors; not all types of impacts are included, such as reputational damage and physical losses.
Erola et al. [41]	Post-attack impact quantification technique, using Conditional Value at Risk (CVaR) by identifying and categorising assets, threats, and control effectiveness, determining incident impacts, simulating threat occurrences and resulting losses, and calculating the CVaR from the loss distribution.	The effectiveness of this technique hinges on significant advancements in data quality and availability, more sophisticated threat modelling approaches, integration of machine learning for dynamic risk assessment and the incorporation of human behaviour factors.
Franco et al. [42]	Post-attack impact quantification technique, using Real Cyber Value at Risk (RCVaR), leveraging industry reports by collecting and scaling data, calculating economic impact considering company costs, time value of money, and industry factors, and determining the probability of higher cost incurrence.	The effectiveness of this technique hinges on validating the RCVaR with more real-world scenarios, refinement of the factor calibration process for organisational tailoring, scaling cost distribution variance, and exploration of machine learning techniques for enhanced estimation accuracy.
Facchinetti et al. [63]	Post-attack impact quantification technique by creating an ordinal dataset of business lines, incorporating event types with three severity levels, calculating a criticality index based on severity and business line/event type combinations, and aggregating risk levels across business lines or event types.	This technique’s use of ordinal data limits its ability to derive capital buffers for cyber risk coverage to quantify the economic impact of cyber attacks, as could be done using quantitative data. Ordinal data cannot be used to create a taxonomy to support quantification of cyber attack impact
Goel et al. [65]	Post-attack impact quantification technique using event-study analysis to interpret the influence of security breaches on stock prices, manifesting as statistically negative abnormal returns (AR) and cumulative abnormal returns (CAR) around breach announcements.	This technique’s event-study analysis results could be inaccurate due to difficulties in defining the event and its exact timing, difficulties in choosing the appropriate event window and the choice of the model used to calculate abnormal returns.
Greenfield et al. [34]	Post-attack impact quantification technique, by constructing a business model of cyber attack activity, evaluating the severity and incidence of harms, prioritising harms based on severity, and establishing causality.	This framework is limited by its qualitative approach to quantification, the lack of a mechanism for comparing harm across different classes of bearers, and the presence of normative dimensions.
Kamiya et al. [69]	Post-attack impact quantification technique using event study from news articles to calculate abnormal returns models and analysing stock price changes around the announcement date.	The limitation of this technique is that the out-of-pocket cost may be underestimated, which tends to introduce inaccuracies in the model results.
Lazrag et al. [16]	Post-attack impact quantification technique using Business Logic Modeling(BLM) for operational impact assessment and its generation using a combination of a resource dependency model and a mission dependency model.	The limitation of using Business Logic Modeling is that it limits the entire scope to only operational cyber security impacts, missing out on other classes of cyber security impact.
Orlando et al. [48]	Post-attack impact quantification technique using Cyber Value at Risk (Cy-VaR), by modelling the frequency (using distributions like Poisson or negative binomial) and severity (using distributions like log-normal, skew-normal, or generalised Pareto) of cyber attacks, potentially using copulas to capture their dependence.	This technique is limited by a lack of standardised security maturity metrics, subjective asset valuation (especially for intangible assets), difficulty estimating attack probabilities due to limited data and unpredictable attacker behaviour, and challenges in understanding interdependencies between system security.
Pollmeier et al. [71]	Post-attack impact quantification technique using hypothetical cyber incident scenarios groups to categorise cyber security impact, explicitly defining those impact categories, and developing corresponding quantification metrics.	Its broadly defined incident–impact relationship and qualitative case study methodology hinder generalisability, inability to measure reputational cost, and the need for further testing and evaluation of its proposed structure, categorisations, and metrics.
Portela et al. [72]	Post-attack impact quantification technique using a scenario-based case study involving data retrieval from media sources, timeline construction, estimation of activity drops and direct costs, loss distribution parameter estimation, and sensitivity analysis to illustrate potential cost impacts on health institutions.	The technique’s accuracy is limited by a lack of public data on the respective health sector’s cyber attacks, reliance on a hypothetical scenario, a focus on direct costs excluding indirect and long-term impacts, and simplified assumptions in the sensitivity analysis.
Razavi et al. [74]	Post-attack impact quantification technique by collecting transaction data from various bank systems, statistically analysing the data, diagnosing downtime, estimating lost transactions, calculating the associated financial loss, and determining operational risks and downtime costs.	This technique’s limitation is that it focuses solely on denial-of-service attacks, neglecting other cyber attack types and broader cost considerations like reputational damage, customer behaviour changes, and the role of cyber insurance.
Tahmasebi et al. [77]	Post-attack impact quantification technique utilising data from industry-leading reports such as Verizon’s 2023 Data Breach Investigations Report and IBM’s Cost of a Data Breach Report 2023.	The paper does not include a model to calculate the multidimensional impact of cyber attacks. It does not include a model to verify the data adopted from other data sources.
Thomas et al. [78]	Post-attack impact quantification technique using an eight-step process involving scope definition, evidence identification, timeline construction, impact indicator listing, uncertainty identification, branching activity modelling, cost function parameter estimation, and aggregate impact estimation via Monte Carlo simulation to quantify the impact of cyber attacks.	It assumes that holistic impact is more meaningful than asset-level impact, prioritising estimation of recovery activities over vulnerability/exploit enumeration and assuming insignificant losses untranslatable to recovery, further needing the development of tailored model selection and innovative techniques for estimating branching probabilities.
Zadeh et al. [43]	Post-attack impact quantification utilising the Breach Level Index (BLI), severity based on weighted variables, including record count, data type, breach source, and malicious use. This involved data acquisition using the BLI formula on S and P 500 breaches, data modelling by classifying breaches by type, records, location, and circumstances, and finally, data analysis applying the BLI formula.	The limitation of this technique is that it does not determine whether organisations significantly alter security measures post-breach, absorb the consequences, and maintain existing practices. Additionally, the study’s scope is limited by its focus on intentional or externally caused breaches, excluding accidental breaches due to human or programming errors, and its analysis of only S and P 500 firms.

**Table 11:** Data sources for quantifying the multidimensional impact of cyber attack.

Data Source(s)	The Usage	Limitations
Operational Riskdata eXchange [84]	It supplies data variables, including loss frequency data (e.g., average loss per event), severity (e.g., largest losses), event types (e.g., external fraud) and loss categories (e.g., cyber-related operational risk) used for historical simulation of past cyber attacks [32,39].	VaR computation can be significantly inaccurate due to underestimation of tail risk and misleading precision (i.e., a single VaR figure).
Privacy Rights Clearinghouse [85]	It supplies data variables, including the number of records (N, e.g., 130 million), type of data (t, e.g., PII assigned a weight of 1), source of the breach (s, e.g., hacking assigned a weight of 4), and whether or not the data has been used for nefarious purposes (A, e.g., no immediate action assigned a weight of 1). All weighted values are assigned based on the Stiennon scale [43] and are plugged into a logarithm to the base of 10 function BLI = log10(N * t * s * A), with the resulting score the Breach Level Index (BLI) [43,69].	BLI computation can be inaccurate because of insufficient information on breaches, lacking BLI detailed inputs, outdated information, subjectivity in weight assignment, and geographical biases.
Incident Response Metrics Database	It supplies data variables, including the estimated number of affected individuals (e.g., ranging from 10,000 to 50,000), the types of data compromised (e.g., PII), the potential attack vectors (e.g., malware), and the potential business impact scenarios (e.g., regulatory fines). These variables are then inputted into the Monte Carlo simulation, to generate a distribution of aggregate impact values. The generated values reflect a range of possible outcomes for cyber attack impact [78].	Branching activity model and indicators impact computation can be limited because most organisations do not have this level of data, and when available, they are incomplete, inaccurate, and unreliable.
Banking Transactions	It supplied data variables, including the number of banking transactions made across the Point of Sale(PoS), Internet/Mobile banking and Automated Teller Machines (ATM) for purchases, withdrawals, transfers, charges, and balance requests over five years. This data is used to model yearly patterns/numbers of transactions to detect deviations that may suggest Denial of Service (DoS) attacks. The income per lost transaction is computed as the financial impact of the DoS attack [74].	DoS financial impact cost calculated based on banking transaction data can be significantly inaccurate due to the streamlined assumption that all lost transactions are due to DoS attacks.
Industry Reports (e.g., Accenture and Ponemon Institue LLC [86], and Cost of a Data Breach Report 2024 [8])	They supply data variables including the valuation of the company at a specific year (e.g., 10 billion dollars), the discounted valuation of the company (e.g., 2 billion dollars), the coefficient of variation ratio (e.g., 0.8), the discount cost (e.g., 12 percent ), and a set of 11 parameter ratios (e.g., ranging from 0.05 to 0.2, representing various risk factors). These values are plugged into a mathematical formula. The resulting company cost year value represents the Relative Change in Value at Risk (RCVar) [42,47].	RCVar computation could be complex due to the scarcity of publicly available and rich data related to cyber attacks and breaches, which is a well-known challenge for cyber security approaches [70]
Advisen Dataset [87], Internal Asset Database, Security Control Inventory and Risk Assessment Reports.	They supply, input variables including asset (e.g., email system), asset value (e.g., 10,000 dollars), threat (e.g., phishing), threat probability (e.g., 0.02 percent), controls (phishing software with residual risk of 0.7) and harms (e.g., data breach value of 20,000 dollars, and 0.02 probability). The data is plugged into a Monte Carlo simulation for all assets, determining which threats occur and which controls fail and calculating the resulting losses and harm propagation to determine which harms are realised, considering control effectiveness at each step. The output of the data distribution gives the Cyber Value at risk (CVar) [41].	CVar computation could be inconsistent due to insufficient data (i.e., quality and quantity) to train and validate the CVar. This insufficient data could make the computed value of CVar inaccurate.
Technology News/Websites (CNET [88] and ZDNET [89] and LexisNexis [90])	They supply data variables, including private companies listed on the stock exchange market, their dividends, earnings, and mergers/acquisitions on the day before the data breach announcement, the day after the data breach announcement, and the day after the data breach announcement. This data is plugged into an event study analysis to understand the impact of the security breach on the company’s market value using a logarithm function [58].	The limitation of this data source is it relies on assumption of efficient markets and rational investors, which may not always hold true.

**Table 12:** Evaluation Metrics for Cyber security Risk Quantification with ML Algorithms.

Citation	Use Cases	Machine Learning Algorithms				Evaluation Criteria			
		**SVM**	**NB**	**CNN**	**LSTM**	**Accuracy**	**Recall**	**Precision**	**F1 Score**
Abdulsatar et al. [50]	Assessment of cyber risk	**X**	X	X	X	**✓**	**✓**	**✓**	**✓**
Ahmadi-Assalemi et al. [51]	Modelling anomalous behaviour	**✓**	**✓**	X	X	**✓**	**✓**	**✓**	**✓**
Alagappan et al. [52]	Estimating financial impact	X	**✓**	X	X	**✓**	**✓**	**✓**	**✓**
Ali et al. [53]	Assessment of cyber risk	**✓**	**✓**	**✓**	**✓**	**✓**	**✓**	**✓**	**✓**
Alsaadi et al. [54]	Estimating financial impact	X	X	**✓**	**✓**	**✓**	**✓**	**✓**	**✓**
Biswas et al. [57]	Estimating insurance premium	**✓**	**✓**	X	X	**✓**	**✓**	**✓**	**✓**
Franco et al. [64]	Assessment of cyber risk	**✓**	X	X	X	X	**✓**	**✓**	**✓**
Goyal et al. [67]	Predicting cyber attack	X	X	X	**✓**	X	**✓**	**✓**	**✓**
Kalin et al. [68]	Assessment of cyber risk	X	X	X	X	**✓**	X	X	X
Kumar et al. [47]	Estimating economic impact	X	**✓**	X	X	X	X	X	X
Rafaiani et al. [73]	Assessment of risk exposure	**✓**	X	X	X	**✓**	X	X	X
Wickramasinghe Anuja [79]	Assessment of cyber risk	**✓**	X	**✓**	X	**✓**	**✓**	**✓**	**✓**
Yao et al. [81]	Predicting cyber risk	X	**✓**	X	X	X	X	X	X

## Data Availability

All data used in this study are available upon reasonable request. Please get in touch with the authors to obtain access.
